# Evidence-based interventions for identifying candidate quality indicators to assess quality of care in diabetic foot clinics: a scoping review

**DOI:** 10.1186/s12889-024-18306-2

**Published:** 2024-04-10

**Authors:** Flora Mbela Lusendi, An-Sofie Vanherwegen, Kris Doggen, Frank Nobels, Giovanni Arnoldo Matricali

**Affiliations:** 1 Health Services Research, Sciensano, Rue Juliette Wytsmanstraat 14, Brussels, 1050 Belgium; 2 Department of Development and Regeneration, KU Leuven, Leuven, Belgium; 3 Multidisciplinary Diabetic Foot Clinic, Onze-Lieve-Vrouwziekenhuis, Aalst, Belgium; 4 Multidisciplinary Diabetic Foot Clinic, University Hospital Leuven, Leuven, Belgium

**Keywords:** Diabetic foot ulcer, Quality of healthcare, Quality indicators, Evidence-based medicine, Health service research

## Abstract

**Background:**

Foot ulcers in people with diabetes are a serious complication requiring a complex management and have a high societal impact. Quality monitoring systems to optimize diabetic foot care exist, but a formal and more evidence-based approach to develop quality indicators (QIs) is lacking. We aimed to identify a set of candidate indicators for diabetic foot care by adopting an evidence-based methodology.

**Methods:**

A systematic search was conducted across four academic databases: PubMed, Embase CINAHL and Cochrane Library. Studies that reported evidence-based interventions related to organization or delivery of diabetic foot care were searched. Data from the eligible studies were summarized and used to formulate process and structure indicators. The evidence for each candidate QI was described in a methodical and transparent manner. The review process was reported according to the “Preferred Reported Items for Systematic reviews and Meta-Analysis” (PRISMA) statements and its extension for scoping reviews.

**Results:**

In total, 981 full-text articles were screened, and 322 clinical studies were used to formulate 42 candidate QIs.

**Conclusions:**

An evidence-based approach could be used to select candidate indicators for diabetic foot ulcer care, relating to the following domains: wound healing interventions, peripheral artery disease, offloading, secondary prevention, and interventions related to organization of care. In a further step, the feasibility of the identified set of indicators will be assessed by a multidisciplinary panel of diabetic foot care stakeholders.

**Supplementary Information:**

The online version contains supplementary material available at 10.1186/s12889-024-18306-2.

## Introduction

Diabetic foot ulceration (DFU) is a common disability burden, with a 25% lifetime risk in persons with diabetes [1]; it is estimated that 40 to 60 million people are globally affected by DFU [2]. The condition has an important impact on quality of life of both persons with diabetes and DFU and their informal caregivers [3, 4] and causes substantial healthcare costs [2, 5, 6]. Because of the significant physical, psychosocial and economic impact of diabetic foot disease, there is a global search by the medical community for systems of quality evaluation and monitoring of diabetic foot care [7–9]. The “International Working Group on the Diabetic Foot” (IWGDF) recommends auditing all aspects of diabetic foot care to ensure that clinical practice meets accepted standards of care [10].

The management of DFU is complex and demanding. DFU care requires multidisciplinary collaboration across the healthcare landscape, in an often lengthy care process, in which not only the quality of the care provided by each individual healthcare provider is important, but also the quality of the collaboration and of the overall organization of the care.

Quality monitoring of such complex care is equally demanding. It requires several quality of care indicators (QIs) that describe the performance that should occur for a particular type of patient or the related health outcomes, followed by the assessment of whether patients’ care is consistent with the indicators based on evidence-based standards of care [11]. QIs can be related to structure, process or outcome of healthcare [12] and/or meet additional quality-of-care frameworks such as the six aims for the “21st Century Health Care System” provided by the Institute of Medicine [13]. In order to be useful, they must be developed, tested and implemented with scientific rigor. For a care process to be considered as a valid QI, it must have been demonstrated to be associated with a desired outcome. Similarly, a structure of care can be used as QI, if it increases the likelihood of a desired outcome or of a process, which improves an outcome. Further, for outcome indicators to be valid, variations in outcomes must be attributable to variations in care quality [14]. Two key steps have been emphasized for developing QIs: the synthesis of information from a variety of sources (e.g. literature, clinical data) and a validated method to determine the extent to which experts agree about the proposed set of indicators [15].

In diabetic foot care, there already exist some national initiatives on quality evaluation and monitoring. Belgium, Germany and the United Kingdom (UK) have issued national quality initiatives for accreditation and auditing of diabetic foot services [16, 17]. The German Working group on the Diabetic Foot developed a certification procedure for diabetic foot centers that includes data collection on structure of care and on limited parameters of process of care (e.g. vascular intervention) and outcome (e.g. rate of minor and major amputations) [7, 18]. These indicators were defined by an expert board within the working group. In Belgium, indicators were developed by Belgian diabetic foot experts and used in the context of a nationwide quality initiative, named IQED-Foot (Initiative for Quality improvement and Epidemiology in multidisciplinary Diabetic Foot Clinics). A large number of QIs are related to processes of care (e.g. revascularization of ischemic lower limbs) and to outcomes (e.g. ulcer healing rate) [19]. No indicators of structure of care are used, as only diabetic foot clinics (DFCs) that meet the national requirements for accreditation participate in the quality evaluation. In addition, the UK launched a “National Diabetes Foot Care Audit”, based on a pilot project that assessed methodology for the measurement of processes and outcomes in the management of diabetic foot ulcers using QIs defined by a national working group [8]. It included indicators related to diabetes management, ulcer outcome but also patient-reported outcome measures.

Although the data collections in the context of these audits are valuable, they have a number of shortcomings that need to be addressed. The QIs used differ from one initiative to another, and do not cover all aspects of care. The current indicators are largely based on expert opinion, without a systematic search of the literature nor any formal consensus among diabetic foot care stakeholders.

Therefore, there is a need for a more systematic and evidence-based approach to develop QIs for diabetic foot care. So far, a detailed methodology describing the identification of QIs in diabetic foot care has not been published. The purpose of this study was to perform a systematic and open-minded (i.e. not limited to guidelines) search of the literature on evidence-based interventions that could be used as process or structure indicators to assess quality in DFCs. The result of this work represented the first key step in developing a set of evidence-based QIs that will be used to achieve consensus among diabetic foot care stakeholders.

## Methods

This scoping review was conducted to provide an overview of the available scientific evidence. The review process was reported according to the “Preferred Reported Items for Systematic reviews and Meta-Analysis” (PRISMA) statements [20] and its extension for scoping reviews [21]. The results of the scoping review aim to be used to formulate a set of candidate quality indicators, which are evaluated by a diabetic foot care stakeholder panel during a modified Delphi consensus.

### Search strategy

We searched for systematic reviews and primary clinical studies to identify aspects of the organization of care (structure) or delivery of care (process) that could be defined as quality of care indicators. The topics “foot ulcer” or “amputation” combined with the topic “diabetes mellitus” were used to build the search strategy for four electronic databases: PubMed, Embase, CINAHL and Cochrane Library. Controlled terms from Medical Subject Headings (MeSH) in PubMed and Cochrane Library, from Emtree in Embase.com and from CINAHL Headings in CINAHL were used in the search query. An additional file shows the search query in detail (See Additional file 1). We focused on producing a search strategy that was sensitive. To do so, we use more general terms, whilst avoiding specific search terms related to “quality of care” in order to not miss potentially eligible studies. In addition, a lot of research on the effectiveness of interventions do not phrase their results in terms of "quality of care", but simply in terms of improving outcomes. The following publication types were excluded from the search strategy: letter, editorial, comment, case reports, and note. In addition, searches were limited to publications in English, French and Dutch. The search period ran from the inception of the databases to March 03, 2020.

### Inclusion and exclusion criteria

To be eligible, a study had to fulfill all the criteria detailed in Table 1. Because of efficiency concerns, we applied a limitation on publication year. The review team (FML, ASV, KD, FN, GM) decided that the literature review would cover the period from 01/01/2011 to 03/03/2020 based on the assumption that the number of publications on diabetic foot has significantly increased over the last 10 years [22], and that therefore the relevant and up-to-date interventions will have been reviewed during the past 10 years. We searched for publications reporting clinical research studies that evaluated the effect of an intervention on health-related outcomes.

**Table 1:** Detailed description of the inclusion/exclusion criteria

Criteria	Inclusion criteria	Exclusion criteria
**Language**	French, Dutch and English	Any language other than French, Dutch or English
**Publication year**	From 01/01/2011 to 03/03/2020	31/12/2010 or earlier
**Study type**	A clinical research study that evaluates interventions on health-related outcomes, whose full-text article could be retrieved from the KU Leuven Libraries collection with institutional access or whose full report was registered or indexed on the platform ClinicalTrials.gov	1) Case reports, conference abstracts, study protocols, letter, editorial, comment, note 2) A clinical trial registered on the platform ClinicalTrial.gov, whose the status has not been reported as “*completed”*
**Study domain**	Studies reporting interventions that address the following domains of diabetic foot care: • organization of care • wound healing interventions • peripheral artery disease (PAD) • offloading • prevention of foot ulcer in people with diabetes with active or history of foot ulceration (secondary prevention)	Studies reporting interventions that address the following domains of diabetic foot care: • diagnosis and treatment of foot infection (antimicrobial therapy, adjunctive treatment and surgical treatment) • prevention of foot ulcer in people with diabetes without active/history of foot ulceration (primary prevention)
**Study design**	1) Studies designed with a control group (randomized or non-randomized) 2) Systematic reviews of controlled studies, with or without meta-analysis	1) Studies addressing the wound healing interventions or offloading domain which, based on the reported study design, do not provide high quality evidence (level of evidence^a^ > 2) 2) Studies which, based on the reported study design, do not provide quality evidence of at least level 3—e.g. case–control, case series, etc 3) Systematic reviews of a combination of studies with eligible and non-eligible designs 4) Systematic reviews which do not provide a summarized conclusion (pooled results or general statements) about the effect of the intervention
**Population**	1) People with diabetes: • with active diabetic foot ulceration (DFU) or history of DFU, it includes the different stages of the complication: critical limb ischemia (CLI)—infection/osteomyelitis—gangrene • having surgical wounds subsequent to a DFU (post-operative wound) 2) Mixed or more comprehensive study population (e.g. chronic wounds, PAD patients) where the eligible study population is specifically studied	1) People with diabetes (non-exhaustive list): with Charcot foot, venous ulcer, claudication, amputation not due to a DFU, acute limb ischemia, etc 2) Mixed or more comprehensive study population (e.g. chronic wounds, PAD patients) where the eligible study population was not specifically studied
**Intervention**	Interventions in patients with active or history of DFU at the level of: 1) organization of diabetic foot care or 2) delivery of diabetic foot care (diagnostic, treatment, secondary prevention), measuring a change in outcomes related to the patient or to the foot/limb or to the healthcare costs	1) Interventions which do not fit into the intervention groups extracted from the literature 2) Interventions reported by only one single study (not related to organization of care) 3) Interventions related to the administration of patient-reported outcome instruments
**Outcome**	Quantitative outcomes: 1) related to the foot/limb: ulcer healing, minor amputation, major amputation, change in ulcer area, post-operative wound healing, ulcer recurrence, ulcer incidence 2) related to the patient: survival, amputation-free survival, patient-reported outcome measures (PROMs), quantified care experiences (e.g. PREMs) 3) related to healthcare costs: length of stay, cost-effectiveness, quality-adjusted life year (QALY)	Results for which a measure of the statistical significance is not reported

^a^Level of Evidence provided by Oxford Centre for Evidence-Based Medicine (OCEBM) http://www.cebm.net/wpcontent/uploads/2014/06/CEBM-Levels-of-Evidence-2.1.pdf

We included studies reporting interventions which addressed one of the following chapters covered by the guidelines provided by the IWGDF [23]: interventions to enhance healing of foot ulcers in persons with diabetes (wound healing interventions), peripheral artery disease (PAD), offloading and prevention of foot ulcers in patients with diabetes. Since the success in DFU management also depends on effective organizational features [10], we also covered interventions related to organization of care. We decided to not cover the domain of infection (e.g. antimicrobial therapy, adjunctive treatment and surgical treatment) since two extensive systematic reviews have been performed recently by the IWGDF, leading to updated Guidelines on the diagnosis and treatment of foot infection in persons with diabetes [24, 25]. For the offloading domain, the treatment with “Total Contact Casting” (TCC) was proven to be efficient more than 10 years ago [26–30] and is nowadays commonly used as the gold standard. Therefore, TCC was not included in the evidence-based approach to develop QIs. Moreover, studies exclusively dealing with prevention of foot ulcers in people with diabetes without active or history of foot ulceration (primary prevention) were excluded because it did not inform us about the management of an existing DFU. We also excluded interventions reported by only one single study (not related to organization of care). The main criteria we used were: (i) studies designed with a control group (randomized or non-randomized) or systematic reviews of controlled studies; (ii) inclusion of patients with diabetes and an active or history of foot ulceration (including the different stages of the complication); (iii) description of an intervention related to the organization or delivery of diabetic foot care (diagnostic, treatment, secondary prevention): (iv) measuring change in outcomes related to the foot/limb or to the patient or to the healthcare costs.

### Selection process

Following completion of the database searches, the extracted records were entered into the reference management software Zotero (https://www.zotero.org/). Three researchers (FML, KD, SC) independently merged search results and removed duplicates [31–34]. Then, one researcher (FML) uploaded the resulting records to the online application “Rayyan” [35] (www.rayyan.ai) to help in the assessment of studies. Two researchers (FML, KD) independently and blindly reviewed studies by titles and abstracts to assess their eligibility based on the criteria mentioned above. At several occasions, they met to discuss any disagreements regarding their selections until consensus was obtained. The level of agreement between the two reviewers was assessed by calculating Cohen’s kappa values [36]. The full-texts of records that appeared potentially eligible were retrieved by one reviewer (FML), who was helped by an administrative collaborator (VB). The same reviewer (FML) examined the obtained full-text records. If necessary, other members of the reviewer team (ASV, FN, GM) were consulted to make the final decision.

### Data extraction

Firstly, we collected comprehensive information about each eligible study using a structured form. The following data were extracted: author, year of publication, study design, sample size, ulcer characteristics, the studies’ exclusion criteria, period of follow-up, intervention type, description of intervention, number of patients randomized to each intervention arm, studied outcomes, and whether differences between study groups were statistically significant. The clinical studies were grouped according to the domains listed above. One reviewer (FML) extracted the data and another reviewer (ASV) checked the entered data. Next, we used a second structured form to group studies within each domain based on the intervention types and outcomes studied. For each study, we recorded if the intervention had a significant or a non-significant effect on the reported outcomes and we defined population parameters based on ulcer characteristics. We used this information to generate evidence-based statements.

An evidence-based statement frames the association between an identified intervention and an eligible outcome using the PICO (population, intervention, control and outcome) criteria. The association of intervention-outcome was established based on the set of eligible publications. Lastly, the generated evidence-based statements were used to phrase candidate quality of care indicators. Each candidate indicator was expressed as a proportion, with a given denominator, i.e. the population evaluated by the indicator, and a numerator, i.e. the portion of the denominator that satisfies the condition of the indicator.

### Description of existing supporting evidence

We developed an easy-to-use scoring system to be able to describe the strength of evidence provided by a large amount of identified eligible studies. This allowed us to communicate the certainty of evidence supporting the association between an identified intervention and an outcome.

In this scoring system, we used three factors to determine the quality of a study: the study design, the sample size and the scientific impact of the journal in which the study was published.

1. For determining the quality of the study design we adapted the levels of evidence provided by the Oxford Centre for Evidence-Based Medicine (OCEBM) [37–39] (Table 2).

**Table 2:** Levels of evidence for determining the quality of the study design

	**Domains**	
**Levels of Evidence (LoE)**	**Wound healing** **Offloading**	**Surgical procedures from wound healing domain, PAD, secondary prevention, organization of care**
Level 1	Systematic reviews of randomized controlled trials (RCTs), with or without meta-analysis	
Level 2	Randomized controlled trials Systematic reviews of a combination of RCTs and non-randomized controlled studies, or non-randomized controlled studies only, with or without meta-analysis	
Level 3	Not included	Non-randomized controlled studies: Controlled before-after studies, Interrupted Time-series, prospective cohort studies, retrospective cohort studies (propensity score matched, regression technique)
  We targeted studies that provided high levels of evidence (level 1 or 2). However, because some designs are more difficult to set up for some domains of diabetic foot care, we also allowed level 3 evidence for studies reporting interventions related to organization of care, PAD, surgical procedures to enhance wound healing and secondary prevention, and/or outcomes related to healthcare costs.
2. Regarding the sample size, a cut-off was applied based on a median of participants for a parallel group trial reported by Chan et al. [40] and also adopted by the “CONSORT” guidelines [41]. A sample size of ≥ 32 participants per treatment group was considered as “High”, while a sample size of < 32 participants per treatment group was considered as “Low”.
3. The scientific impact was reported by using the Journal category ranking and quartiles based on the journal’s impact factor and provided by the Journal Citation Reports (JCR) [42] (See Additional file 2). The publication year of the article was used to select the quartile year.

Our scoring system attributed a weight or “evidence score” to each combination of the three criteria. An additional file shows the evidence score value attributed based on the three criteria (See Additional file 3). The reduction in points was non-linear in order to reflect the impact of each factor on publication quality. Finally, an evidence score was assigned to each study, independent of the statistical significance/non-significance of the reported intervention effect.

Following this, a mean score was calculated for the collection of publications reporting the same intervention, subdivided according to outcome. A separate mean score was calculated for publications reporting a significant effect and publications reporting no significant effect. The certainty of the evidence-based statement was categorized based on the mean score of the collection of publications reporting a significant effect. However, the statement was downgraded by one category in cases where the mean evidence score of the publications reporting no significant effect was equal to or higher than the mean evidence score of the publications reporting a significant effect. An additional file shows the categories of certainty of the evidence-based statements (See Additional file 4).

## Results

### Results of the search

**Fig. 1:**
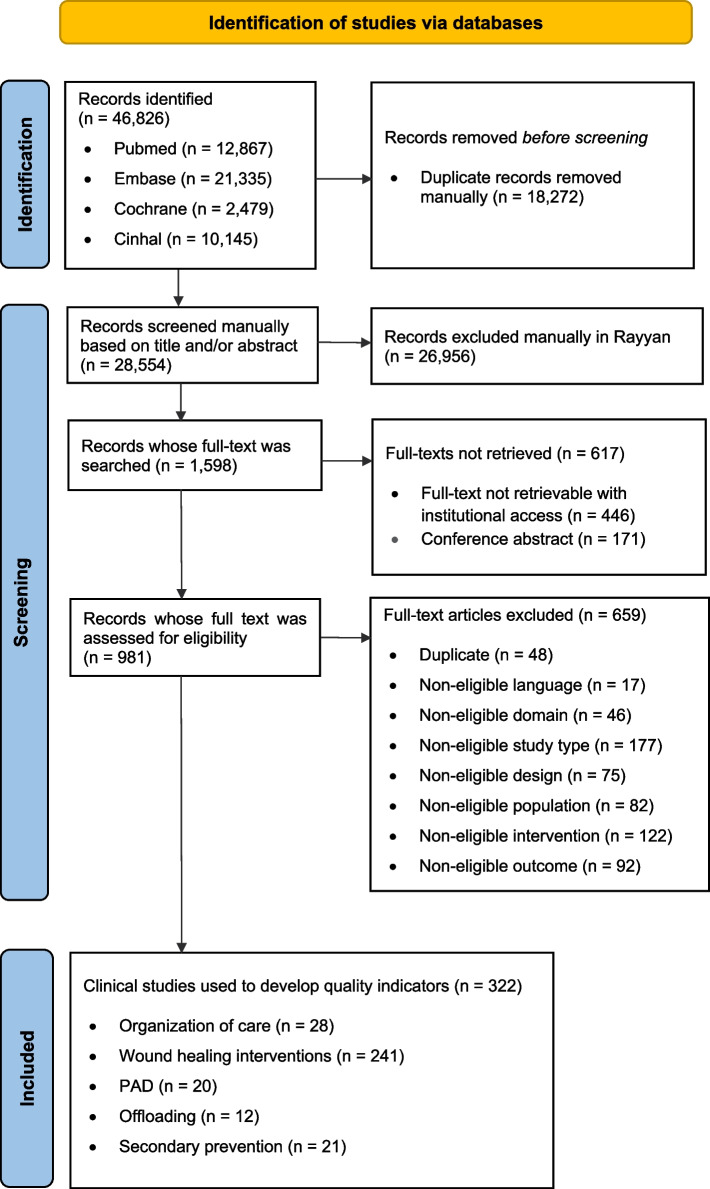
Study selection process and reasons for exclusion based on “PRISMA” flow diagram

The electronic search in online databases yielded a total of 46,826 records. The “PRISMA” flow diagram for the study selection process and reasons for exclusion are shown in Fig. 1. After removal of duplicates and title/abstract screening, 1,598 records from 2011 up to March 2020 were selected for a full-text search. There were 617 records for which the full-text could not be retrieved either because the full-text was not retrievable from the KU Leuven Libraries collection with institutional access or because they were conference abstracts. We assessed 981 full-text articles for eligibility. A total of 322 clinical studies met our inclusion criteria and were used to develop candidate QIs. We excluded 659 of the assessed full-texts, most often because a detailed inspection showed that the publication did not report a clinical study that evaluates an intervention (non-eligible study type, *n* = 177). Numerous studies were also ineligible because the results for outcomes of interest and/or a measure of statistical significance were not reported (non-eligible outcome, *n* = 92). A series of publications were excluded because of the reported type of intervention (non-eligible intervention, *n* = 122); these were: interventions (not related to organization of care) supported by an only one single study, surgical procedures with another aim than revascularization, offloading, debridement or amputation, investigation of a single revascularization technique without control group, interventions based on natural agents only available in some areas (e.g. Chinese herbals, Papaya pulp dressing, Topical Kiwifruit), interventions outside of conventional clinical settings (e.g. home monitoring tools or telemedicine approach). Studies that regarded mixed or more comprehensive population (e.g. chronic wounds, PAD patients) that did not focus on our target population were also excluded (non-eligible study population, *n* = 82). Others reasons for exclusion were the following: study designs which did not provide the expected level of evidence (non-eligible study design, *n* = 75), the reported intervention was related to the infection domain (non-eligible domain, *n* = 46), records were identified as duplicate after having checked the content of their full-text (duplicate, *n* = 48), retrieved full-text was not in an eligible language although an English abstract was previously found (non-eligible language, *n* = 17).

### Included studies and evaluated interventions

The eligible clinical studies evaluated several types of interventions (see the references of included studies in Additional file 5). We defined subcategories for most intervention groups to better represent our findings. Among the 28 studies that addressed the *organization of care domain*, the following intervention groups were listed: introduction of multidisciplinary foot care, integration of a podiatric specialty in the multidisciplinary foot care team, implementation of a care management program for diabetic foot, implementation of a Pay-for-Performance program, implementation of nurse-led care. A large majority of studies (*n* = 241) covered the *wound healing intervention domain* and evaluated the following interventions: non-biological dressings (2 subcategories: non-biological dressing impregnated with antimicrobial agents, non-biological dressing not impregnated with antimicrobial agents), bioengineered skin substitutes (3 subcategories: acellular dermal matrix, allogeneic skin substitute, autologous skin substitute), isolated cellular therapy, hyperbaric oxygen therapy (HBOT) (3 subcategories according to the patient perfusion status: not specified, adequate or inadequate), isolated growth factor, negative pressure wound therapy (NPWT), physical therapy (4 subcategories: laser/phototherapy, extracorporeal shockwave therapy, ultrasound therapy, physical therapy other than laser, shockwave or ultrasound), gas therapy (2 subcategories: topical oxygen therapy, ozone therapy or combined oxygen-ozone therapy), nutritional supplementation (2 subcategories: single nutrient supplementation, multi-nutrient supplementation), pharmacological agents (2 subcategories: action on vessels, action on immunity), debridement (2 subcategories: biological, enzymatic) and non-revascularization surgical procedures (3 subcategories: amputation, bony surgical offloading, soft tissue surgical offloading). The studies addressing the *PAD domain* (*n* = 20) compared endovascular surgery and bypass surgery or evaluated the revascularization based on the angiosome concept. Among studies addressing the *offloading domain* (*n* = 12), some evaluated offloading performed with non-removable knee-high offloading devices in comparison to offloading performed with removable knee-high offloading devices whilst others evaluated offloading performed with knee-high offloading devices in comparison to offloading performed with ankle-high devices. The studies related to the *secondary prevention domain* included 3 types of interventions (*n* = 21): patient education, footwear and/or insoles (2 subcategories: therapeutic footwear and/or custom-made insoles, or custom-made shoes with and without optimization by plantar pressure measurements) and the application of a prevention management program.

### Summary of evidence

In a nutshell, the potential beneficial effect of interventions related to *organization of care* on DFU outcomes was supported by low evidence. The evidence that indicates that interventions related to the *wound healing intervention domain* may have a beneficial effect on DFU outcomes was heterogeneous. Overall, a possible beneficial effect on ulcer healing by treatment with non-biological dressings not impregnated with antimicrobial agents, bioengineered skin substitutes, isolated cellular therapy, isolated growth factors and NWPT was supported by moderate to high evidence. Unlike treatment with laser/phototherapy, extracorporeal shockwave therapy, topical oxygen therapy or enzymatic debridement, the possible beneficial effect on ulcer healing by treatment with ozone therapy or combined oxygen-ozone therapy, single nutrient supplementation, pharmacological agents having action on immunity, or biological debridement was supported by low evidence.

In the *PAD domain*, low evidence indicates that revascularization with endovascular surgery compared to open vascular surgery may have a beneficial effect on limb salvage/amputation-free survival and amputation events. The same certainty of evidence was observed the other way around, when comparing revascularization with open vascular surgery to endovascular surgery. No studies were identified from the literature search with no revascularization as control group. Concerning the *offloading domain*, very high evidence indicates that non-removable knee-high offloading devices may have a beneficial impact on time to healing, when compared to removable knee-high offloading devices. In the *secondary prevention domain*, the effect of patient education was the most studied, but the evidence indicating a potential beneficial effect on diverse DFU outcomes was low. A complete overview of the evidence supporting the extracted interventions from the literature is available in Additional file 5.

### Candidate evidence-based indicators

A total of 42 candidate evidence-based QIs for studying quality of care in DFCs were developed from our findings from existing literature. An overview is presented in Table 3. They were attributed to the level of care (hospital, national) and the aspect of care addressed (structure, process or outcome).

**Table 3:** List of quality indicators per domain, developed from evidence-based interventions identified through a scoping review

**Domain: Organization of care**					
**Intervention**		**Indicator**	**Numerator/Denominator**	**Level of care**	**Indicator type**
**Introduction of multidisciplinary foot care**	1	Proportion of people with a diabetic foot ulcer receiving multidisciplinary foot care	**Numerator**: The number of people with a diabetic foot ulcer receiving multidisciplinary foot care **Denominator**: The total number of people with a diabetic foot ulcer	Hospital National	Structure
**Integration of a podiatric specialty in the multidisciplinary foot care team**	2	Proportion of people with diabetic foot ulcer receiving multidisciplinary foot care with an integrated podiatric specialty	**Numerator**: The number of people with a diabetic foot ulcer receiving multidisciplinary foot care with an integrated podiatric specialty **Denominator**: The total number of people with a diabetic foot ulcer		
**Implementation of a care management program for diabetic foot**	3	Proportion of people with a diabetic foot ulcer treated within the context of a care management program for diabetic foot	**Numerator**: The number of people with a diabetic foot ulcer treated within the context of a care management program for diabetic foot **Denominator**: The total number of people with a diabetic foot ulcer	National	
**Implementation of a Pay-for-Performance program**	4	Proportion of diabetic foot clinics that participate to a pay-for-performance program	**Numerator**: The number of diabetic foot clinics that participate to a pay-for-performance program **Denominator**: The total number of diabetic foot clinics	National	
**Implementation of nurse-led care**	5	Proportion of people with a diabetic foot ulcer receiving nurse-led care	**Numerator**: The number of people with a diabetic foot ulcer receiving nurse-led care **Denominator**: The total number of people with a diabetic foot ulcer	Hospital National	
**Domain: Wound healing interventions**					
**Intervention**		**Indicator**	**Numerator/Denominator**	**Level of care**	**Indicator type**
**Treatment with non-biological dressings**	6	Proportion of people with a non-healing diabetic foot ulcer treated with non-biological dressings (umbrella indicator^a^)	**Numerator**: The number of people with a non-healing diabetic foot ulcer treated with any kind of non-biological dressing **Denominator**: The total number of people with a non-healing diabetic foot ulcer	Hospital National	Process
	7	Proportion of people with a non-healing diabetic foot ulcer treated with non-biological dressings impregnated with antimicrobial agents^b^	**Numerator**: The number of people with a non-healing diabetic foot ulcer treated with non-biological dressing impregnated with antimicrobial agents **Denominator**: The total number of people with a non-healing diabetic foot ulcer		
	8	Proportion of people with a non-healing diabetic foot ulcer treated with non-biological dressings not impregnated with antimicrobial agents^b^	**Numerator**: The number of people with a non-healing diabetic foot ulcer treated with non-biological dressing not impregnated with antimicrobial agents **Denominator**: The total number of people with a non-healing diabetic foot ulcer		
**Treatment with bioengineered skin substitutes**	9	Proportion of people with a non-healing diabetic foot ulcer treated with a bioengineered skin substitutes (umbrella indicator^a^)	**Numerator**: The number of people with a non-healing diabetic foot ulcer treated with at least one type of bioengineered skin substitutes **Denominator**: The total number of people with a non-healing diabetic foot ulcer		
	10	Proportion of people with a non-healing diabetic foot ulcer treated with acellular dermal matrix	**Numerator**: The number of people with a non-healing diabetic foot ulcer treated with acellular dermal matrix **Denominator**: The total number of people with a non-healing diabetic foot ulcer		
	11	Proportion of people with a non-healing diabetic foot ulcer treated with allogeneic skin substitute	**Numerator**: The number of people with a non-healing diabetic foot ulcer treated with allogeneic skin substitute **Denominator**: The total number of people with a non-healing diabetic foot ulcer		
	12	Proportion of people with a non-healing diabetic foot ulcer treated with autologous skin substitute	**Numerator**: The number of people with a non-healing diabetic foot ulcer treated with autologous skin substitute **Denominator**: The total number of people with a non-healing diabetic foot ulcer		
**Treatment with isolated cellular therapy**	13	Proportion of people with a non-healing diabetic foot ulcer treated with isolated cellular therapy	**Numerator**: The number of people with a non-healing diabetic foot ulcer treated with isolated cellular therapy **Denominator**: The total number of people with a non-healing diabetic foot ulcer	Hospital National	Process
**Treatment with hyperbaric oxygen therapy**	14	Proportion of people with diabetic foot ulcer treated with systemic hyperbaric oxygen therapy	**Numerator**: The number of people with a diabetic foot ulcer treated with systemic hyperbaric oxygen therapy **Denominator**: The total number of people with a diabetic foot ulcer		
	15	Proportion of people with a diabetic foot ulcer and adequate perfusion treated with systemic hyperbaric oxygen therapy	**Numerator**: The number of people with a diabetic foot ulcer and adequate perfusion treated with systemic hyperbaric oxygen therapy **Denominator**: The total number of people with a diabetic foot ulcer and adequate perfusion		
	16	Proportion of people with diabetic foot ulcer and inadequate perfusion treated with systemic hyperbaric oxygen therapy	**Numerator**: The number of people with a diabetic foot ulcer and an inadequate perfusion treated with systemic hyperbaric oxygen therapy **Denominator**: The total number of people with a diabetic foot ulcer and an inadequate perfusion		
**Treatment with isolated growth factor**	17	Proportion of people with a non-healing diabetic foot ulcer treated with isolated growth factor	**Numerator**: The number of people with a non-healing diabetic foot ulcer treated with isolated growth factor **Denominator**: The total number of people with a non-healing diabetic foot ulcer		
**Treatment with negative pressure wound therapy**	18	Proportion of people with a non-healing diabetic foot ulcer treated with negative pressure wound therapy	**Numerator**: The number of people with a non-healing diabetic foot ulcer treated with negative pressure wound therapy **Denominator**: The total number of people with a non-healing diabetic foot ulcer		
**Treatment with physical therapy**	19	Proportion of people with a non-healing diabetic foot ulcer treated with laser/phototherapy	**Numerator**: Proportion of people with a non-healing diabetic foot ulcer treated with laser/phototherapy **Denominator**: The total number of people with a non-healing diabetic foot ulcer	Hospital National	Process
	20	Proportion of people with a non-healing diabetic foot ulcer treated with extracorporeal shockwave therapy	**Numerator**: The number of people with a non-healing diabetic foot ulcer treated with extracorporeal shockwave therapy **Denominator**: The total number of people with a non-healing diabetic foot ulcer		
	21	Proportion of people with a non-healing diabetic foot ulcer treated with ultrasound therapy	**Numerator**: The number of people with a non-healing diabetic foot ulcer treated with ultrasound therapy **Denominator**: The total number of people with a non-healing diabetic foot ulcer		
	22	Proportion of people with a non-healing diabetic foot ulcer treated with physical therapy other than laser, shockwave or ultrasound	**Numerator**: The number of people with a non-healing diabetic foot ulcer treated with physical therapy other than laser, shockwave or ultrasound **Denominator**: The total number of people with a non-healing diabetic foot ulcer		
**Treatment with gas therapy**	23	Proportion of people with a non-healing diabetic foot ulcer treated with topical oxygen therapy	**Numerator**: The number of people with a non-healing diabetic foot ulcer treated with topical oxygen therapy **Denominator**: The total number of people with a non-healing diabetic foot ulcer		
	24	Proportion of people with a non-healing diabetic foot ulcer treated with ozone therapy or combined oxygen-ozone therapy	**Numerator**: The number of people with a non-healing diabetic foot ulcer treated with ozone therapy or combined oxygen-ozone therapy **Denominator**: The total number of people with a non-healing diabetic foot ulcer		
**Treatment with nutritional supplementation**	25	Proportion of people with a non-healing diabetic foot ulcer treated with a single nutrient supplementation	**Numerator**: The number of people with a non-healing diabetic foot ulcer treated with a single nutrient supplementation **Denominator**: The total number of people with a non-healing diabetic foot ulcer	Hospital National	Process
	26	Proportion of people with a non-healing diabetic foot ulcer treated with a multi-nutrient supplementation	**Numerator**: The number of people with a non-healing diabetic foot ulcer treated with multi-nutrient supplementation **Denominator**: The total number of people with a non-healing diabetic foot ulcer		
**Treatment with pharmacological agents**	27	Proportion of people with a non-healing diabetic foot ulcer treated with pharmacological agents having an action on vessels	**Numerator**: The number of people with a non-healing diabetic foot ulcer treated with pharmacological agents having an action on vessel **Denominator**: The total number of people with a non-healing diabetic foot ulcer		
	28	Proportion of people with a non-healing diabetic foot ulcer treated with pharmacological agents having an action on immunity	**Numerator**: The number of people with a non-healing diabetic foot ulcer treated with pharmacological agents having an action on immunity **Denominator**: The total number of people with a non-healing diabetic foot ulcer		
**Treatment with debridement**	29	Proportion of people with a non-healing diabetic foot ulcer treated with biological debridement	**Numerator**: The number of people with a non-healing diabetic foot ulcer treated with biological debridement **Denominator**: The total number of people with a non-healing diabetic foot ulcer		
	30	Proportion of people with a non-healing diabetic foot ulcer treated with enzymatic debridement	**Numerator**: The number of people with a non-healing diabetic foot ulcer treated with enzymatic debridement **Denominator**: The total number of people with a non-healing diabetic foot ulcer		
**Treatment with surgical procedures**	31	Proportion of people with a non-healing diabetic foot ulcer treated with amputation	**Numerator**: The number of people with a non-healing diabetic foot ulcer treated with amputation **Denominator**: The total number of people with a non-healing diabetic foot ulcer	Hospital National	Process
	32	Proportion of people with a non-healing diabetic foot ulcer treated with bony surgical offloading	**Numerator**: The number of people with a non-healing diabetic foot ulcer treated with bony surgical offloading **Denominator**: The total number of people with a non-healing diabetic foot ulcer		
	33	Proportion of people with a non-healing diabetic foot ulcer treated with soft tissue surgical offloading	**Numerator**: The number of people with a non-healing diabetic foot ulcer treated with soft tissue surgical offloading **Denominator**: The total number of people with a non-healing diabetic foot ulcer		
**Domain: Peripheral artery Disease (PAD)**					
**Revascularization**	34	Proportion of people with diabetic foot ulcer and inadequate perfusion treated with endovascular surgery	**Numerator**: The number of people with a diabetic foot ulcer and inadequate perfusion treated with endovascular surgery **Denominator**: The total number of people with a diabetic foot ulcer and inadequate perfusion	Hospital National	Process
	35	Proportion of people with diabetic foot ulcer and inadequate perfusion treated with open vascular surgery	**Numerator**: The number of people with a diabetic foot ulcer and inadequate perfusion treated with open vascular surgery **Denominator**: The total number of people with a diabetic foot ulcer and inadequate perfusion		
	36	Proportion of people with diabetic foot ulcer and inadequate perfusion undergoing revascularization based on the angiosome concept	**Numerator**: The number of people with a diabetic foot ulcer and inadequate perfusion undergoing revascularization based on the angiosome concept **Denominator**: The total number of people with a diabetic foot ulcer and inadequate perfusion		
**Domain: Offloading**					
**Intervention**		**Indicator**	**Numerator/Denominator**	**Level of care**	**Indicator type**
**Offloading with non-removable knee-high devices**	37	Proportion of people with a non-infected, non-ischemic plantar neuropathic diabetic foot ulcer treated with a non-removable knee-high offloading device	**Numerator**: The number of people with a non-infected, non-ischemic plantar neuropathic diabetic foot ulcer treated with a non-removable knee-high offloading device **Denominator**: The total number of people with a non-infected, non-ischemic plantar neuropathic diabetic foot ulcer	Hospital National	Process
**Offloading with knee-high offloading devices**	38	Proportion of people with a non-infected, non-ischemic plantar neuropathic diabetic foot ulcer treated with a knee-high offloading device	**Numerator**: The number of people with a non-infected, non-ischemic plantar neuropathic diabetic foot ulcer treated with a knee-high offloading device **Denominator**: The total number of people with a non-infected, non-ischemic plantar neuropathic diabetic foot ulcer		
**Domain: Secondary prevention**					
**Patient education**	39	Proportion of people with a (history of) diabetic foot ulcer receiving patient education	**Numerator**: The number of people with a (history of) diabetic foot ulcer receiving patient education **Denominator**: The total number of people with a (history of) diabetic foot ulcer	Hospital National	Process
**Footwear and/or insoles**	40	Proportion of people with a history of peripheral neuropathy (PNP) receiving therapeutic footwear and/or custom-made insoles, or custom-made shoes	**Numerator**: The number of people with a history of PNP receiving therapeutic footwear and/or custom-made insoles, or custom-made shoes **Denominator**: The total number of people with a history of PNP		
	41	Proportion of people with a (history of) diabetic foot ulcer receiving optimization by plantar pressure measurements of their custom-made footwear and/or insoles	**Numerator**: The number of people with a (history of) diabetic foot ulcer receiving optimization by plantar pressure measurements of their custom-made footwear and/or insoles **Denominator**: The total number of people with a (history of) diabetic foot ulcer		
**Protocol-driven multidisciplinary prevention**	42	Proportion of people with a (history of) diabetic foot ulcer treated within the context of a prevention management program for diabetic foot	**Numerator**: The number of people with a (history of) diabetic foot ulcer treated within the context of a prevention management program for diabetic foot **Denominator**: The total number of people with a (history of) diabetic foot ulcer		

^a^Umbrella indicator = unifying indicator under which the specific and related interventions was grouped and which allows to assess the delivery of such therapy regardless the type

^b^Honey derivatives, silver or antibiotics

## Discussion

There is a need for a more evidence-based approach in the development of QIs for diabetic foot care. In this study, we adopted a systematic approach to search for evidence-based interventions from the existing literature and to formulate, based on an evaluation of our search findings, evidence-based candidate QIs on the structures and processes of care. It is not our intention to displace existing, deeply rooted QIs, but to propose additional candidate indicators in an evidence-based manner that can reinforce existing indicators. This evidence-based approach does not take into account clinical relevance or feasibility. We therefore consider this a first step in which possible indicators are collected for which good evidence exists, and then in a next step a stakeholder panel will decide which indicators are useful and feasible for implementation in quality monitoring.

Our evidence-based selection approach resulted in the collection of 42 candidate QIs, including 5 structure indicators and 37 process indicators. Although we only based our methodology on clinical studies, not on guidelines, our resulting candidate QIs span the majority of domains defined by the IWGDF guidelines [10]. Among these are several well-known process indicators, already in use in ongoing quality promotion initiatives (Belgium, Germany, UK), but we also proposed several additional indicators. Our indicators included a larger range of interventions and covered several topics that are not used in many quality evaluation systems and for which clinical interest has been growing. Examples are, nutritional status [43, 44], use of lipid-lowering therapy [45], and of new therapies like cellular therapies [46] or topical oxygen therapy [47]. Despite the fact that for some of these candidate indicators no randomized controlled trials are available (or feasible), these processes are already part of clinical practice and could receive attention as QIs during the evaluation by a stakeholder panel.

In the domain of organization of care we selected indicators commonly reported in the literature such as the establishment of a multidisciplinary team approach or the integration of podiatric care but also less frequent indicators such as the implementation of protocolized care or of pay-for-performance, not implemented by most DFCs so far [16].

In our review, interventions on patient health-related quality of life (QoL) were not included, although the assessment of the patient well-being and function through patient-reported outcome instruments is already proposed as process of care indicator in the UK [8]. This might be explained by the fact this domain is still in full development. Literature that investigates the relationship between psychological interventions and DFU outcomes is still scarce [48], and thus too limited to be able to make evidence based recommendations on QIs.

We did not aim to generate outcome of care indicators in this study because they are already considered as an important goal in diabetic foot care. Besides, the methodology to identify and validate such QIs differs from the approach used in this study. It requires adjustment for differences in case mix and other external factors to ensure fair comparisons among institutions or physicians [49, 50].

The availability of good quality studies providing high level of evidence was limited for topics such as organization of care or surgical procedures. Recently, proposals have been formulated to produce higher quality studies in the PAD domain [51, 52]. Conversely, numerous studies with high evidence were found to support the indicators addressing wound healing interventions and more particularly new therapies like bioengineered skin substitutes or isolated cellular therapy. This can be attributed to the great expansion observed for this body of research over the last decade. Nevertheless, practical concerns could arise in using these wound care procedures as quality indicators in routine care. For instance, issues may rise regarding the storage of such products that requires specific conditions to maintain cell viability. Another challenge may be related to their varied effects and high cost, making it difficult for clinicians to determine which product is appropriate for the patient. This is a clear example of candidate QIs that need the next step of evaluation by a stakeholder panel to decide if they are feasible for implementation in quality monitoring.

Our detailed methodology contributes to the field by using clinical studies as primary sources for possible quality measurements rather than guidelines, predominantly used for the development of QIs so far [53]. A practical guideline presents a framework for optimal care in the context of complex medical decision-making. However, it may reflect the views of the stakeholders involved in its development and quality measures that can be derived from it may be limited in scope. Our open-minded systematic search in the literature helped to identify domains and indicators of quality of care that are not (yet) considered by expert panels. In addition, we have listed the scientific evidence for each candidate QI in a methodical, precise and transparent manner. We developed an easy-to-use scoring system, based on objective criteria, to be able to describe the strength of evidence provided by a large amount of identified eligible studies in an easy to understand format for a stakeholder panel that need to judge on the feasibility of the candidate indicators. The fact that we did not use the standard systems commonly used for assessing certainty of evidence could be seen as a limitation. Yet, this is mainly due to the purpose of our study. We did not need to apply detailed criteria such as heterogeneity or publication bias because our aim was not to judge about the estimate of an effect [54].

We conducted a literature review to provide an exhaustive overview of the existing evidence that demonstrates the linkage between an intervention and an outcome, and thus the possible use of that intervention as a structure or process indicator to assess quality in DFCs. In a next step, the described evidence will be used as a supportive element in order to guide a stakeholder panel in their selection of appropriate QIs. Furthermore, if we were to use standard systems, we would have to use several tools to fit to the heterogeneous encountered designs, which will have made our work more complicated, considering the number of studies that we included.

We have limited ourselves to articles from the last 10 years, to keep the number of articles under review feasible, but also to reflect the current practice in DFCs. However, we strongly realize that the evidence for several pre-existing QIs is based on older literature and do not question it. An example is the use of TCC as a gold standard for offloading. A further limitation of our study is that a single review author examined the full-texts of the selected articles, conducted data extraction and rated the evidence. Because these tasks were not conducted dually and independently, we may have introduced some risk of errors. Nonetheless, a large number of records were assessed during the abstract/title phase, which have been performed independently by two reviewers. The calculation of inter-rater reliability (Cohen’s kappa value) indicated an adequate agreement between the 2 reviewers, which increased the reliability of the selected records used for the next selection steps. Full-texts were assessed using straightforward criteria and the reviewer team was frequently consulted to check the plausibility of the decision.

In conclusion, we showed that it is possible to select a set of candidate indicators for diabetic foot care in an evidence-based manner, independently of expert opinion. In this way, various indicators emerged that are not commonly used in quality evaluation of diabetic foot care. In a next step, the identified set of candidate indicators are aimed to be assessed for relevance and practical usefulness by a broad stakeholder panel from all levels of diabetes foot care. A formal methodology needs to be used to stimulate the discussion and measure the collective opinion in an objective way [55]. In a later stage, it will be recommended to perform an impact analysis to evaluate whether implementation of these QIs changes processes of care and improves patient outcomes and/or reduces costs [15]. Furthermore, the update of these QIs will be monitored based on the evolving DFU care needs.

### Supplementary Information

**Supplementary material 1.**

**Supplementary material 2.**

**Supplementary material 3.**

**Supplementary material 4.**

**Supplementary material 5.**

## Data Availability

Data collated and summarized from this review are available from the corresponding author upon reasonable request.
