# Supplementary material for: Evidence-based interventions for identifying candidate quality indicators to assess quality of care in diabetic foot clinics: a scoping review
Source: BMC Public Health. 2024 Apr 10;24:996. doi: 10.1186/s12889-024-18306-2 (PMC11005120; doi:10.1186/s12889-024-18306-2)
Supplement: Supplementary file 1 — Supplementary material 1. [file 12889_2024_18306_MOESM1_ESM.docx]

**Additional table 1. Search strategy**

|  | **In Pubmed** | **In Embase** | **In Cochrane** | **In Cinahl** |
| --- | --- | --- | --- | --- |
| #1 | “Foot Ulcer” [Mesh] | 'foot ulcer'/exp | [mh "Foot Ulcer"] | MH "Foot Ulcer+" |
| #2 | "Amputation” [MeSH Terms:NoExp] | 'below knee amputation'/exp | [mh ^Amputation] | (MH "Below knee amputation")  OR  (MH "Above knee amputation")) |
| #3 | foot-ulcer* [tiab] | ‘foot ulcer*’:ti,ab,kw | (foot NEXT ulcer*): ti,ab,kw | TI ("foot ulcer*")  OR  AB ("foot ulcer*") |
| #4 | plantar-ulcer* [tiab] | 'plantar-ulcer'/exp  OR  'plantar-ulcer*':ti,ab,kw | (plantar NEXT ulcer*): ti,ab,kw | TI ( "plantar ulcer*")  OR  AB ( "plantar ulcer*") |
| #5 | amput* [tiab] | 'amput*':ti,ab,kw | (amput*): ti,ab,kw | TI ("amput*")  OR  AB ("amput*") |
| #6 | "diabetes mellitus" [MeSH Terms] | 'diabetes mellitus'/exp | [mh " diabetes mellitus"] | (MH diabetes+) |
| #7 | diabet* [tiab] | 'diabet*': ti,ab,kw | (diabet*): ti,ab,kw | TI (diabet*)  OR  AB (diabet*) |
| #8 | diabetic-foot [tiab] | diabetic foot'/exp OR ‘diabetic foot’:ti,ab,kw | ("diabetic foot"):ti,ab,kw | TI ("diabetic foot")  OR  AB ("diabetic foot") |
| #9 | diabetic-feet [tiab] | ‘diabetic feet’:ti,ab,kw | ("diabetic feet"):ti,ab,kw | TI ("diabetic feet")  OR  AB ("diabetic feet") |
| #10 | Letter [Publication Type] | ‘Letter’/exp |  | PT (Letter) |
| #11 | Editorial [Publication Type] | ‘Editorial’/exp |  | PT (Editorial) |
| #12 | Comment [Publication Type] | ‘Note’/exp |  | PT (Commentary) |
| #13 | Case-reports [Publication Type] |  |  |  |
| #14 | #1 OR #2 OR #3 OR #4 OR #5 | #1 OR #2 OR #3 OR #4 OR #5 | #1 OR #2 OR #3 OR #4  #5 | #1 OR #2 OR #3 OR #4  #5 |
| #15 | #6 OR #7 | #6 OR #7 | #6 OR #7 | #6 OR #7 |
| #16 | #8 OR #9 | #8 OR #9 | #8 OR #9 | #8 OR #9 |
| #17 | #10 OR #11 OR #12 OR #13 | #10 OR #11 OR #12 |  | #10 OR #11 OR #12 |
| #18 | #14 AND #15 | #14 AND #15 | #14 AND #15 | #14 AND #15 |
| #19 | #18 OR #16 | #18 OR #16 | #18 OR #16 | #18 OR #16 |
| #20 | #19 NOT#17 | #19 NOT#17 |  | #19 NOT#17 |
| #21 | #20 Filters: Publications in Dutch, English, French | #20 AND ([dutch]/lim  OR [english]/lim  OR [french]/lim) |  | #20 Narrow by Language : dutch/flemish, english, french |
