# Supplementary material for: Evidence-based interventions for identifying candidate quality indicators to assess quality of care in diabetic foot clinics: a scoping review
Source: BMC Public Health. 2024 Apr 10;24:996. doi: 10.1186/s12889-024-18306-2 (PMC11005120; doi:10.1186/s12889-024-18306-2)
Supplement: Supplementary file 2 — Supplementary material 2. [file 12889_2024_18306_MOESM2_ESM.docx]

**Additional table 2. Journal category ranking and quartiles based on the journal’s impact factor**

| **Category rankings** | **Quartiles** |
| --- | --- |
| Q1 | Top 25% |
| Q2 | Between top 25% and 50% |
| Q3 | Between 50% and 75% |
| Q4 | Bottom 25% |
