# Supplementary material for: Evidence-based interventions for identifying candidate quality indicators to assess quality of care in diabetic foot clinics: a scoping review
Source: BMC Public Health. 2024 Apr 10;24:996. doi: 10.1186/s12889-024-18306-2 (PMC11005120; doi:10.1186/s12889-024-18306-2)
Supplement: Supplementary file 3 — Supplementary material 3. [file 12889_2024_18306_MOESM3_ESM.docx]

**Additional table 3. Scoring system attributing an evidence score value to each eligible study based on the level of evidence, sample size and scientific impact**

| **Evidence score** | **Level of Evidence** | **Sample Size** | **Scientific Impact** |
| --- | --- | --- | --- |
| 10 | I | High | Q1 |
| 9 | I | Low | Q1 |
| 9 | I | High | Q2 |
| 8 | I | Low | Q2 |
| 8 | I | High | Q3 |
| 7 | I | Low | Q3 |
| 7 | I | High | Q4 |
| 6 | I | Low | Q4 |
| 8 | II | High | Q1 |
| 7 | II | Low | Q1 |
| 7 | II | High | Q2 |
| 6 | II | Low | Q2 |
| 6 | II | High | Q3 |
| 5 | II | Low | Q3 |
| 5 | II | High | Q4 |
| 4 | II | Low | Q4 |
| 5 | III | High | Q1 |
| 4 | III | Low | Q1 |
| 4 | III | High | Q2 |
| 3 | III | Low | Q2 |
| 3 | III | High | Q3 |
| 2 | III | Low | Q3 |
| 2 | III | High | Q4 |
| 1 | III | Low | Q4 |
