# Supplementary material for: Evidence-based interventions for identifying candidate quality indicators to assess quality of care in diabetic foot clinics: a scoping review
Source: BMC Public Health. 2024 Apr 10;24:996. doi: 10.1186/s12889-024-18306-2 (PMC11005120; doi:10.1186/s12889-024-18306-2)
Supplement: Supplementary file 4 — Supplementary material 4. [file 12889_2024_18306_MOESM4_ESM.docx]

**Additional table 4. Categories of certainty of the evidence-based statements based on the mean of evidence scores**

| **Mean evidence score** | **Evidence statement category** |
| --- | --- |
| ≥ 9 | There is very high evidence in literature that intervention (I) may... |
| ≥ 8 and < 9 | There is high evidence in literature that intervention (I) may... |
| ≥ 7 and < 8 | There is good evidence in literature that intervention (I) may... |
| ≥ 6 and < 7 | There is moderate evidence in literature that intervention (I) may... |
| < 6 | There is low evidence in literature that intervention (I) may... |
