# Supplementary material for: Evidence-based interventions for identifying candidate quality indicators to assess quality of care in diabetic foot clinics: a scoping review
Source: BMC Public Health. 2024 Apr 10;24:996. doi: 10.1186/s12889-024-18306-2 (PMC11005120; doi:10.1186/s12889-024-18306-2)
Supplement: Supplementary file 5 — Supplementary material 5. [file 12889_2024_18306_MOESM5_ESM.docx]

**Additional table 5. Overview of the evidence supporting the extracted interventions from the literature**

The certainty of the evidence supporting the association between an intervention and an outcome is indicated.

The studies reporting a significant beneficial effect on outcome are in black, while the studies reporting

a detrimental or no significant effect are in grey.

| **Supporting studies** | **Mean**  **evidence score** | **Certainty of**  **evidence-based**  **statements** | **References** |
| --- | --- | --- | --- |
| **ORGANIZATION OF CARE DOMAIN** | | | |
| **Introduction of multidisciplinary foot care** | | | |
| **Ulcer healing** | | | |
| 1 study (III) | 4 | Low | (1) |
| 1 study (III) | 3 |  | (2) |
| **Major amputation events** | | | |
| 2 studies (II) - 9 studies (III) | 4 | Low | (1,3–12) |
| 3 studies (III) | 4 |  | (2,13,14) |
| **Minor amputations** |  |  | |
| 4 studies (III) | 4 | Low | (1,9,13,15) |
| 2 studies (III) | 2,5 |  | (2,10) |
| **Length of hospital stay** |  |  | |
| 3 studies (III) | 4 | Low | (9,13,15) |
| 2 studies (III) | 3,5 |  | (2,14) |
| **Integration of a podiatric specialty in the multidisciplinary foot care team** | | | |
| **Ulcer healing** | | | |
| 2 studies (III) | 2,5 | Low | (16,17) |
| **Major amputation events** | | | |
| 5 studies (III) | 4 | Low | (17–21) |
| **Minor amputations** | | | |
| 1 study (III) | 5 | Low | (18) |
| **Length of hospital stay** | | | |
| 1 study (III) | 4 | Low | (22) |
| **Implementation of a care management program** | | | |
| **Ulcer healing** | | | |
| 1 study (III) | 4 | Low | (23) |
| 1 study (III) | 3 |  | (24) |
| **Major amputation events** | | | |
| 3 studies (III) | 4 | Low | (23–25) |
| 1 study (III) | 2 |  | (26) |
| **Minor amputations** | | | |
| 1 study (III) | 3 | Low | (25) |
| **Mortality** | | | |
| 3 studies (III) | 3 | Low | (23–25) |
| **Cost per patients** | | | |
| 1 study (III) | 3 | Low | (25) |
| **Implementation of a Pay-for-Performance program** | | | |
| **Non-traumatic lower extremity amputation events** | | | |
| 1 study (III) | 4 | Low | (27) |
| **Implementation of nurse-led care** | | | |
| **Patient-reported Experience Measures (PREMs)** | | | |
| 1 study (II) | 6 | Moderate | (28) |
| **WOUND HEALING INTERVENTION DOMAIN** | | | |
| **Treatment with non-biological dressings impregnated with antimicrobial agents** | | | |
| **Ulcer healing** | | | |
| 2 studies (I) - 4 studies (II) | 6,5 | Low | (29–34) |
| 2 studies (I) - 5 studies (II) | 6,5 |  | (32)(35–40) |
| **Ulcer area reduction** | | | |
| 3 studies (II) | 4, | Low | (33,37,41) |
| 2 studies (II) | 5,5 |  | (42,43) |
| **Time to healing** | | | |
| 2 studies (II) | 6 | Moderate | (30,39) |
| 2 studies (II) | 5,5 |  | (37,43) |
| **Stump healing** | | | |
| 1 study (II) | 6 | Moderate | (44) |

Additional table 5 (continued)

| **Treatment with non-biological dressings not impregnated with antimicrobial agents** | | | |
| --- | --- | --- | --- |
| **Ulcer healing** | | | |
| 9 studies (I) - 6 studies (II) | 8 | High | (34,45–58) |
| 3 studies (II) | 6 |  | (59–61) |
| **Ulcer area reduction** | | | |
| 9 studies (II) | 5 | Low | (47,50,62–68) |
| **Time to healing** | | | |
| 6 studies (II) | 6,6 | Moderate | (45–47,50,51,69) |
| **Cost-effectiveness** | | | |
| 1 study (I) - 1 study (III) | 5 | Low | (49,70) |
| 1 study (III ) | 2 |  | (71) |
| **Hospitalization days** | | | |
| 2 studies (III) | 2,5 | Low | (72,73) |
| 1 study (III) | 2 |  | (72) |
| **Treatment with bioengineered skin substitutes: acellular dermal matrix** | | | |
| **Ulcer healing^a^** | | | |
| 2 studies (I) - 3 studies (II) | 7,2 | Moderate | (74–78) |
| 1 studies (I) - 2 studies (II) | 7,6 |  | (79–81) |
| **Ulcer healing at 6 weeks** | | | |
| 1 study (I) | 9 | Very high | (82) |
| **Ulcer healing at 12 weeks** | | | |
| 1 study (I) - 2 studies (II) | 7 | Good | (82–84) |
| **Ulcer healing at 16 weeks** | | | |
| 1 study (I) - 2 studies (II) | 7 | Good | (82,85,86) |
| **Ulcer area reduction** | |  | |
| 3 studies (II) | 6 | Moderate | (78,79,87) |
| **Time to healing** | | | |
| 1 study (I) - 1 study (II) | 8 | High | (82,83) |
| 1 study (II) | 7 |  | (81) |
| **Ulcer recurrence** | | | |
| 1 study (II) | 7 | Moderate | (81) |
| 1 study (II) | 7 |  | (86) |
| **Amputation events** | | | |
| 1 study (I) - 1 study (II) | 7,5 | Good | (75,80) |
| **Quality of life** | | | |
| 1 study (II) | 7 | Good | (86) |
| **Treatment with bioengineered skin substitutes: allogenic skin substitute** | | | |
| **Ulcer healing**^a^ | | | |
| 4 studies (I) - 6 studies (II) | 7 | Moderate | (74,80,88–95) |
| 1 studies (I) - 1 study (II) | 7,5 |  | (95,96) |
| **Ulcer healing at 6 weeks** | | | |
| 1 study (I) - 4 studies (II) | 7 | Good | (97–101) |
| **Ulcer healing at 12 weeks** | | | |
| 1 studies (I) - 8 studies (II) | 7 | Good | (96–100,102–105) |
| **Ulcer healing at 16 weeks** | | | |
| 1 study (II) | 7 | Good | (103) |
| **Probability of ulcer healing** | | | |
| 1 study (I) - 5 studies (II) | 6,8 | Moderate | (88,93,103,104,106,107) |
| **Ulcer recurrence** | |  | |
| 1 study (II) | 6 | Low | (108) |
| 1 study (II) | 7 |  | (93) |
| **Ulcer area reduction at 16 weeks** | | | |
| 5 studies (II) | 6,2 | Moderate | (91,94,100,105,109) |
| 1 study (II) | 6 |  | (110) |
| **Amputation events** | | | |
| 1 study (I) - 1 study (II) | 7,5 | Good | (80,108) |
| 1 study (II)^b^ | 4 |  | (91) |

Additional table 5 (continued)

| **Time to healing** | | | |
| --- | --- | --- | --- |
| 2 studies (I) - 9 studies (II) | 6,7 | Moderate | (88,89,92–94,97,99,100,104,106,111) |
| 1 study (II) | 6 |  | (96) |
| **Cost-effectiveness** | | | |
| 1 study (I) - 3 studies (II) -  2 studies (III) | 5,8 | Low | (102,104,110,112–114) |
| **Treatment with bioengineered skin substitutes: autologous skin substitute** | | | |
| **Ulcer healing^a^** | | | |
| 3 studies (II) | 7,6 | Good | (115–117) |
| 1 study (II) | 6 |  | (118) |
| **Ulcer healing at 12 weeks** | | | |
| 1 study (II) | 5 | Low | (96) |
| 1 study (II) | 6 |  | (118) |
| **Probability of ulcer healing** | | | |
| 1 study (II) | 8 | High | (115) |
| **Ulcer area reduction** | | | |
| 2 studies (II) | 7 | Good | (117,118) |
| 1 study (II) | 5 |  | (96) |
| **Time to healing** | | | |
| 3 studies (II) | 6,6 | Moderate | (96,116,117) |
| 1 study (II) | 6 |  | (118) |
| **Treatment with isolated cellular therapy** | | | |
| **Ulcer healing** | | | |
| 1 study (I) - 1 study (II) | 7,5 | Good | (119,120) |
| 1 study (II) | 7 |  | (121) |
| **Ulcer area reduction** | | | |
| 1 study (II) | 5 | Low | (122) |
| **Time to healing** | | | |
| 1 study (I) | 9 | Very high | (123) |
| 1 study (II) | 7 |  | (124) |
| **Reduction of pain** | | | |
| 1 study (I) | 9 | Very high | (119) |
| **Reduction of amputation events** | | | |
| 1 study (I) - 1 study (II) | 7,5 | Good | (119,120) |
| **Treatment with systematic hyperbaric oxygen in people with DFU** | | | |
| **Ulcer healing^c^** | | | |
| 2 studies (I) - 6 studies (II) | 7,25 | Moderate | (125–131) |
| 2 study (I) - 3 studies (II) | 8 |  | (131–135) |
| 1 study (III) ^d^ | 5 |  | (136) |
| **Ulcer area reduction** | | | |
| 2 studies (I) - 2 studies (II) | 6,75 | Low | (134,135,137,138) |
| 1 study (I) - 1 study (II) | 7 |  | (128,139) |
| **Reduction of major amputation events^c^** | | | |
| 2 studies (I) - 4 studies (II) | 8 | Moderate | (125,129–131,133) |
| 3 studies (I) - 2 studies (II) | 8,2 |  | (131,132,134,135,139) |
| 1 study (III) ^d^ | 5 |  | (136) |
| **Reduction of minor amputation events** | | | |
| 1 study (I) - 1 study (II) | 7 | Moderate | (125,131) |
| 3 studies (I) - 2 studies (II) | 8,4 |  | (130,131,134,135,139) |
| **Quality of life** | | | |
| 3 studies (II) | 5,6 | Low | (127,138,140) |
| 1 study (II) | 7 |  | (141) |
| **Cost-effectiveness** | | | |
| 1 study (I) - 1 study (III) | 3,5 | Low | (128,142) |

Additional table 5 (continued)

| **Treatment with systematic hyperbaric oxygen in people with DFU**  **and adequate perfusion** | | | |
| --- | --- | --- | --- |
| **Ulcer healing** | | | |
| 1 study (II) | 5 | Low | (126) |
| 1 study (III)^d^ | 5 |  | (136) |
| **Treatment with systematic hyperbaric oxygen in people with DFU**  **and inadequate perfusion** | | | |
| **Ulcer healing** | | | |
| 1 study (I) - 1 study (II) | 8 | Good | (125,130) |
| 1 study (II) | 8 |  | (133) |
| **Ulcer area reduction** | | | |
| 1 study (II) | 4 | Low | (137) |
| **Reduction of major amputation events** | | | |
| 1 study (I) - 2 studies (II) | 8 | High | (125,130,133) |
| **Reduction of minor amputation events** | | | |
| 1 study (I) - 1 study (II) | 8 | High | (125,130) |
| **Treatment with isolated growth factors** | | | |
| **Ulcer healing** | | | |
| 11 studies (I) - 6 studies (II) | 8,6 | High | (143–159) |
| 2 studies (I) | 8 |  | (160,161) |
| **Ulcer area reduction** | | | |
| 1 study (I) - 4 studies (II) | 6,8 | Low | (143,149,150,155,162) |
| 3 studies (I) - 3 studies (II) | 6,8 |  | (153,154,160,163–165) |
| **Time to healing** | | | |
| 3 studies (I) - 6 studies (II) | 7 | Moderate | (144,150,151,153,154,159,166–168) |
| 1 study (I) | 10 |  | (155) |
| **Reduction of amputation events** | | | |
| 1 study (I) - 1 study (II) | 7 | Moderate | (161,165) |
| 1 study (I) - 1 study (II) | 7,5 |  | (155,169) |
| **Cost-effectiveness** | | | |
| 4 studies (III) | 3 | Low | (170–173) |
| 1 study (III) | 5 |  | (174) |
| **Treatment with negative pressure wound therapy** | | | |
| **Ulcer healing** | | | |
| 5 studies (I) - 2 studies (II) | 7,8 | Good | (175–181) |
| 1 study (II) | 7 |  | (182) |
| **Ulcer area reduction** | | | |
| 4 studies (I) - 5 studies (II) | 6,1 | Moderate | (175,177–180,183–186) |
| 1 study (I) | 6 |  | (187) |
| **Time to healing** | | | |
| 3 studies (I) - 1 study (II) | 7,7 | Good | (175,177,179,180) |
| 1 study (II) | 4 |  | (186) |
| **Reduction of amputation events** | | | |
| 2 studies (I) - 2 studies (II) | 7,5 | Good | (176,177,185,188) |
| 1 study (I) | 6 |  | (187) |
| **Quality of life** | | | |
| 1 study (II) | 4 | Low | (189) |
| **Cost-effectiveness** | | | |
| 4 studies (III) | 3,25 | Low | (190–193) |
| **Treatment with physical therapy: laser/phototherapy** | | | |
| **Ulcer healing** | | | |
| 3 studies (I) | 9,6 | Very high | (194–196) |
| 2 studies (II) | 5 |  | (197,198) |
| **Ulcer area reduction** | | | |
| 2 studies (I) - 12 studies (II) | 5,9 | Low | (194,195,197,199–209) |
| 1 study (II) | 6 |  | (210) |

Additional table 5 (continued)

| **Treatment with physical therapy: extracorporeal shockwave therapy** | | | |
| --- | --- | --- | --- |
| **Ulcer healing** | | | |
| 2 studies (I) - 2 studies (II) | 7,25 | Good | (211–214) |
| **Ulcer area reduction** |  |  |  |
| 1 study (I) - 2 studies (II) | 6,6 | Low | (211,213,215) |
| 2 studies (I) - 1 study (II) | 7 |  | (211,212,216) |
| **Time to healing** |  |  |  |
| 1 study (II) | 6 | Moderate | (217) |
| **Treatment with physical therapy: ultrasound therapy** | | | |
| **Ulcer area reduction** | | | |
| 3 studies (II) | 5,6 | Low | (218–220) |
| 2 studies (II) | 4 |  | (221,222) |
| **Time to healing** | | | |
| 1 study (II) | 5 | Low | (218) |
| **Treatment with physical therapy: other than laser, shockwave or ultrasound** | | | |
| **Ulcer area reduction** | | | |
| 1 study (I) - 3 studies (II) | 6,5 | Moderate | (223–226) |
| 1 study (II) | 6 |  | (227) |
| **Treatment with gases therapy: topical oxygen therapy** | | | |
| **Ulcer healing** | | |  |
| 3 studies (II) | 6 | Moderate | (228–230) |
| 1 study (II) | 5 |  | (231) |
| **Ulcer area reduction** | | | |
| 3 studies (II) | 6 | Moderate | (228,230,232) |
| **Time to healing** | | | |
| 1 study (II) | 5 | Low | (229) |
| **Treatment with gases therapy: ozone therapy or combined oxygen-ozone therapy** | | | |
| **Ulcer healing** | | | |
| 1 study (II) | 6 | Low | (233) |
| 1 study (I) - 1 study (II) | 7,5 |  | (234,235) |
| **Ulcer area reduction** | | | |
| 1 study (II) | 6 | Low | (233) |
| 1 study (I) - 1 study (II) | 7,5 |  | (234,235) |
| **Time to healing** | | | |
| 1 study (II) | 6 | Moderate | (236) |
| **Amputation events** | | | |
| 1 study (II) | 6 | Moderate | (236) |
| **Treatment with nutritional supplementation: a single nutrient supplementation** | | | |
| **Ulcer healing** | | | |
| 5 studies (II) | 5,8 | Low | (237–241) |
| 1 study (II) | 7 |  | (242) |
| **Treatment with nutritional supplementation: a multi-nutrient nutrient supplementation** | | | |
| **Ulcer area reduction** | | | |
| 1 study (I) - 1 study (II) | 7 | Good | (243,244) |
| **Treatment with pharmacological agents having an action on vessels** | | | |
| **Ulcer area reduction** | | | |
| 2 studies (II) | 5,5 | Low | (245,246) |
| **Quality of life** | | | |
| 1 study (II) | 4 | Low | (247) |
| **Treatment with pharmacological agents having an action on immunity** | | | |
| **Ulcer healing** | | | |
| 2 studies (II) | 5 | Low | (248,249) |
| 1 study (II) | 6 |  | (250) |
| **Ulcer area reduction** | | | |
| 2 studies (II) | 4,5 | Low | (249,251) |
| 1 study (II) | 5 |  | (252) |

Additional table 5 (continued)

| **Treatment with debridement: biological debridement** | | | |
| --- | --- | --- | --- |
| **Ulcer healing** | | | |
| 1 study (II) | 6 | Low | (253) |
| 3 studies (II) | 6,3 |  | (254–256) |
| **Ulcer area reduction** | | | |
| 1 study (II) | 6 | Moderate | (257) |
| **Time to healing** | | | |
| 2 studies (II) | 5,5 | Low | (253,256) |
| 1 study (II) | 6 |  | (255) |
| **Reduction of amputation events** | | | |
| 2 studies (II) | 6,5 | Moderate | (254,256) |
| **Cost-effectiveness** | | |  |
| 2 studies (II) | 6 | Moderate | (255,257) |
| **Treatment with debridement: enzymatic debridement** | | | |
| **Ulcer healing** | | | |
| 1 study (I) | 10 | Very high | (254) |
| **Cost-effectiveness** | | | |
| 1 study (III) | 3 | Low | (258) |
| **Treatment with surgical procedures: amputation** | | | |
| **Reduction of the risk of mortality** | | | |
| 1 study (III) | 3 | Low | (259) |
| 2 studies (III) | 3,5 |  | (260,261) |
| **Beneficial impact on ambulatory function (QoL)** | | | |
| 1 study (III) | 4 | Low | (262) |
| **Treatment with surgical procedures: bony surgical offloading** | | | |
| **Reduction of the risk of mortality** | | | |
| 1 study (III) | 2 | Low | (263) |
| **Time to healing** | | | |
| 3 studies (III) | 1,6 | Low | (263–265) |
| 1 study (III) | 4 |  | (266) |
| **Reduction of amputation events** | | | |
| 2 studies (III) | 1,5 | Low | (265,267) |
| **Ulcer recurrence** | | | |
| 2 studies (III) | 2 | Low | (263,264) |
| 1 study (III) | 1 |  | (265) |
| **Reduction hospitalization rate** | | | |
| 1 study (III) | 2 | Low | (263) |
| **Treatment with surgical procedures: soft tissue surgical offloading** | | | |
| **Ulcer recurrence** | | | |
| 1 study (I) - 1 study (III) | 7 | Moderate | (268,269) |

Additional table 5 (continued)

| **PERIPHERAL ARTERY DISEASE DOMAIN** | | | |
| --- | --- | --- | --- |
| **Revascularization with endovascular surgery (vs. open vascular surgery)** | | | |
| **Limb salvage/amputation-free survival** | | | |
| 2 studies (III) | 2,5 | Low | (270,271) |
| 3 studies (III) | 3 |  | (271–273) |
| **Amputation events** | | | |
| 1 study (III) | 5 | Low | (274) |
| 1 study (II) - 2 studies (III) | 4,6 |  | (272,275,276) |
| **Hospitalization days** | | | |
| 1 study (III) | 3 | Low | (271) |
| 1 study (III) | 5 |  | (277) |
| **Cost-effective** | | | |
| 1 study (III) | 2 | Low | (278) |
| **Revascularization with open vascular surgery (vs. endovascular surgery)** | | | |
| **Limb salvage/amputation-free survival** | | | |
| 3 studies (III) | 4 | Low | (277,279,280) |
| 3 studies (III) | 3 |  | (271–273) |
| **Amputation events** | | | |
| 1 study (III) | 5 | Low | (277) |
| 1 study (II) - 1 study (III) | 5,5 |  | (272,275) |
| **Ulcer healing** | | | |
| 1 study (III) | 5 | Low | (280) |
| **Revascularization based on the angiosome concept** | | | |
| **Limb salvage/amputation-free survival** | | | |
| 1 study (I) - 3 studies (III) | 5,7 | Low | (281–284) |
| 4 studies (III) | 3,7 |  | (282,285–287) |
| **Post-operative wound healing** | | | |
| 1 study (I) -1 study (II) -  5 studies (III) | 4,7 | Low | (281,283–285,287–289) |
| 1 study (III) | 5 |  | (282) |
| **Time to healing** | | | |
| 1 study (III) | 4 | Low | (281) |

Additional table 5 (continued)

| **OFFLOADING DOMAIN** | | | |
| --- | --- | --- | --- |
| **Offloading with non-removable knee-high offloading devices** | | | |
| **Ulcer healing** | | | |
| 3 studies (I) - 3 studies (II) | 7,1 | Moderate | (290–295) |
| 3 studies (I) - 2 studies (II) | 7,6 |  | (291,295–298) |
| **Ulcer area reduction** | | | |
| 1 study (II) | 4 | Low | (292) |
| 1 study (II) | 6 |  | (297) |
| **Time to healing** | | | |
| 1 study (I) | 10 | Very high | (296) |
| 1 study (I) - 3 studies (II) | 6,25 |  | (291,293,297,298) |
| **Offloading with a knee-high offloading devices** | | | |
| **Ulcer healing** | | | |
| 2 studies (I) - 1 study (II) | 7,6 | Good | (290,291,295) |
| 2 studies (I) - 3 studies (II) | 6,8 |  | (290,291,293,299,300) |
| **Time to healing** | | | |
| 2 studies (II) | 6,5 | Moderate | (293,295) |
| 1 study (II) | 6 |  | (300) |
| **Ulcer area reduction** | | | |
| 1 study (II) | 4 | Low | (301) |
| **SECONDARY PREVENTION DOMAIN** | | | |
| **Patient education** | | | |
| **Ulcer incidence** | | | |
| 2 studies (I) - 1 study (II) -  2 studies (III) | 5,8 | Low | (302–306) |
| 2 studies (I) - 1 study (II) | 8 |  | (302,303,307) |
| **Ulcer area reduction** | | | |
| 2 studies (III) | 1 | Low | (308,309) |
| **Quality of life** | | | |
| 1 study (II) - 1 study (III) | 4,5 | Low | (310,311) |
| **Pain** | | | |
| 1 study (II) | 4 | Low | (312) |
| **Length of stay** | | | |
| 1 study (II) | 4 | Low | (312) |
| **Providing therapeutic footwear and/or custom-made insoles, or custom-made shoes** | | | |
| **Plantar ulcer reduction and/or recurrence** | | | |
| 2 studies (I) - 1 study (II) -  1 study (III) | 6,5 | Low | (290,302,313,314) |
| 2 studies (II) | 6,5 |  | (315,316) |
| **Providing optimization by plantar pressure measurements**  **of the custom-made footwear and/or insoles** | | | |
| **Ulcer incidence** | | | |
| 1 study (II) | 8 | High | (317) |
| **Ulcer incidence^e^** | | | |
| 2 studies (II) | 7 | Good | (318,319) |
| **Treatment in the context of a prevention management program** | | | |
| **Treatment cost-effectiveness** | | | |
| 1 study (II) - 2 studies (III) | 4 | Low | (320–322) |

^a^no specific follow-up time was reported

^b^results in favor of control group (not significant)

^c^This outcome is reported in a study (Elraiyah *et al*.) through two levels of evidence

(level of evidence I and II)

^d^results in favor of control group (significant)

^e^but only if a sufficient compliance to wear the footwear is present

**References of supporting studies**

1. Riaz M, Miyan Z, Waris N, Zaidi SIH, Tahir B, Fawwad A, et al. Impact of multidisciplinary foot care team on outcome of diabetic foot ulcer in term of lower extremity amputation at a tertiary care unit in Karachi, Pakistan. Int Wound J. 2019 Jun;16(3):768–72.

2. Coşkun Ö., Uzun G., Karakaş A., Tok D., Çebi G., Çekli Y., et al. The influence of an interdisciplinary diabetic foot team on the outcome of patients with diabetic foot. J Clin Anal Med. 2016;7(4):529–32.

3. Albright RH, Manohar NB, Murillo JF, Kengne LAM, Delgado-Hurtado JJ, Diamond ML, et al. Effectiveness of multidisciplinary care teams in reducing major amputation rate in adults with diabetes: A systematic review & meta-analysis. Diabetes Res Clin Pract. 2020 Jan 11;161:107996.

4. Basiri R, Haverstock BD, Petrasek PF, Manji K. Reduction in Diabetes-Related Major Amputation Rates After Implementation of a Multidisciplinary Model: An Evaluation in Alberta, Canada. J Am Podiatr Med Assoc. 2019 Nov 1;

5. Musuuza J, Sutherland BL, Kurter S, Balasubramanian P, Bartels CM, Brennan MB. A systematic review of multidisciplinary teams to reduce major amputations for patients with diabetic foot ulcers. J Vasc Surg. 2019 Oct 29;

6. Paisey RB, Abbott A, Levenson R, Harrington A, Browne D, Moore J, et al. Diabetes-related major lower limb amputation incidence is strongly related to diabetic foot service provision and improves with enhancement of services: peer review of the South-West of England. Diabet Med J Br Diabet Assoc. 2018;35(1):53–62.

7. Jiménez S, Rubio JA, Álvarez J, Ruiz-Grande F, Medina C. Trends in the incidence of lower limb amputation after implementation of a Multidisciplinary Diabetic Foot Unit. Endocrinol Diabetes Nutr. 2017 Apr;64(4):188–97.

8. Hsu CR, Chang CC, Chen YT, Lin WN, Chen MY. Organization of wound healing services: The impact on lowering the diabetes foot amputation rate in a ten-year review and the importance of early debridement. Diabetes Res Clin Pract. 2015 Jul;109(1):77–84.

9. Lazzarini PA, O’Rourke SR, Russell AW, Derhy PH, Kamp MC. Reduced Incidence of Foot-Related Hospitalisation and Amputation amongst Persons with Diabetes in Queensland, Australia. PloS One. 2015;10(6):e0130609.

10. Martínez-Gómez DA, Moreno-Carrillo MA, Campillo-Soto A, Carrillo-García A, Aguayo-Albasini JL. Reduction in diabetic amputations over 15 years in a defined Spain population. Benefits of a critical pathway approach and multidisciplinary team work. Rev Espanola Quimioter Publicacion Of Soc Espanola Quimioter. 2014 Sep;27(3):170–9.

11. Rubio JA, Aragón-Sánchez J, Jiménez S, Guadalix G, Albarracín A, Salido C, et al. Reducing major lower extremity amputations after the introduction of a multidisciplinary team for the diabetic foot. Int J Low Extrem Wounds. 2014 Mar;13(1):22–6.

12. Alexandrescu V, Hubermont G, Coessens V, Philips Y, Guillaumie B, Ngongang C, et al. Why a multidisciplinary team may represent a key factor for lowering the inferior limb loss rate in diabetic neuro-ischaemic wounds: application in a departmental institution. Acta Chir Belg. 2009 Dec;109(6):694–700.

13. Kim CH, Moon JS, Chung SM, Kong EJ, Park CH, Yoon WS, et al. The Changes of Trends in the Diagnosis and Treatment of Diabetic Foot Ulcer over a 10-Year Period: Single Center Study. Diabetes Metab J. 2018 Aug;42(4):308–19.

14. Plusch D, Penkala S, Dickson HG, Malone M. Primary care referral to multidisciplinary high risk foot services - too few, too late. J Foot Ankle Res. 2015;8:62.

15. Laakso M, Honkasalo M, Kiiski J, Ala-Houhala M, Haapasalo H, Laine HJ, et al. Re-organizing inpatient care saves legs in patients with diabetic foot infections. Diabetes Res Clin Pract. 2017 Mar;125:39–46.

16. Blanchette V, Hains S, Cloutier L. Establishing a multidisciplinary partnership integrating podiatric care into the Quebec public health-care system to improve diabetic foot outcomes: A retrospective cohort. Foot Edinb Scotl. 2019 Mar;38:54–60.

17. Almdal T, Nielsen AA, Nielsen KE, Jørgensen ME, Rasmussen A, Hangaard S, et al. Increased healing in diabetic toe ulcers in a multidisciplinary foot clinic-An observational cohort study. Diabetes Res Clin Pract. 2015 Dec;110(3):315–21.

18. Schmidt BM, Holmes CM, Ye W, Pop-Busui R. A Tale of Two Eras: Mining Big Data from Electronic Health Records to Determine Limb Salvage Rates with Podiatry. Curr Diabetes Rev. 2019;15(6):497–502.

19. Schmidt BM, Wrobel JS, Munson M, Rothenberg G, Holmes CM. Podiatry impact on high-low amputation ratio characteristics: A 16-year retrospective study. Diabetes Res Clin Pract. 2017 Apr;126:272–7.

20. Kröger K, Moysidis T, Feghaly M, Schäfer E, Bufe A, Initiative Chronische Wunden e.V., Germany. Association of diabetic foot care and amputation rates in Germany. Int Wound J. 2016 Oct;13(5):686–91.

21. Gibson TB, Driver VR, Wrobel JS, Christina JR, Bagalman E, DeFrancis R, et al. Podiatrist care and outcomes for patients with diabetes and foot ulcer. Int Wound J. 2014 Dec;11(6):641–8.

22. Cichero MJ, Bower VM, Walsh TP, Yates BJ. Reducing length of stay for acute diabetic foot episodes: employing an extended scope of practice podiatric high-risk foot coordinator in an acute foundation trust hospital. J Foot Ankle Res. 2013 Dec 11;6(1):47.

23. Weck M, Slesaczeck T, Paetzold H, Muench D, Nanning T, von Gagern G, et al. Structured health care for subjects with diabetic foot ulcers results in a reduction of major amputation rates. Cardiovasc Diabetol. 2013 Mar 13;12:45.

24. Setacci C, Sirignano P, Mazzitelli G, Setacci F, Messina G, Galzerano G, et al. Diabetic foot: surgical approach in emergency. Int J Vasc Med. 2013;2013:296169.

25. Tan MLM, Feng J, Gordois A, Wong ESD. Lower extremity amputation prevention in Singapore: economic analysis of results. Singapore Med J. 2011 Sep;52(9):662–8.

26. Rümenapf G, Geiger S, Schneider B, Amendt K, Wilhelm N, Morbach S, et al. Readmissions of patients with diabetes mellitus and foot ulcers after infra-popliteal bypass surgery - attacking the problem by an integrated case management model. VASA Z Gefasskrankheiten. 2013 Jan;42(1):56–67.

27. Sheen YJ, Kung PT, Kuo WY, Chiu LT, Tsai WC. Impact of the pay-for-performance program on lower extremity amputations in patients with diabetes in Taiwan. Medicine (Baltimore). 2018 Oct;97(41):e12759.

28. Nayeri ND, Samadi N, Larijani B, Sayadi L. Effect of nurse-led care on quality of care and level of HbA1C in patients with diabetic foot ulcer: A randomized clinical trial. Wound Repair Regen. 2020;28(3):338–46.

29. Karimi Z, Behnammoghadam M, Rafiei H, Abdi N, Zoladl M, Talebianpoor MS, et al. Impact of olive oil and honey on healing of diabetic foot: a randomized controlled trial. Clin Cosmet Investig Dermatol. 2019;12:347–54.

30. Wang C, Guo M, Zhang N, Wang G. Effectiveness of honey dressing in the treatment of diabetic foot ulcers: A systematic review and meta-analysis. Complement Ther Clin Pract. 2019 Feb;34:123–31.

31. Yakoot M, Abdelatif M, Helmy S. Efficacy of a new local limb salvage treatment for limb-threatening diabetic foot wounds - a randomized controlled study. Diabetes Metab Syndr Obes Targets Ther. 2019;12:1659–65.

32. Kateel R, Adhikari P, Augustine AJ, Ullal S. Topical honey for the treatment of diabetic foot ulcer: A systematic review. Complement Ther Clin Pract. 2016 Aug;24:130–3.

33. Imran M, Hussain MB, Baig M. A Randomized, Controlled Clinical Trial of Honey-Impregnated Dressing for Treating Diabetic Foot Ulcer. J Coll Physicians Surg--Pak JCPSP. 2015 Oct;25(10):721–5.

34. Zhang X, Sun D, Jiang GC. Comparative efficacy of nine different dressings in healing diabetic foot ulcer: A Bayesian network analysis. J Diabetes. 2019 Jun;11(6):418–26.

35. Dumville JC, Lipsky BA, Hoey C, Cruciani M, Fiscon M, Xia J. Topical antimicrobial agents for treating foot ulcers in people with diabetes. Cochrane Database Syst Rev. 2017 14;6:CD011038.

36. Tsang KK, Kwong EWY, To TSS, Chung JWY, Wong TKS. A Pilot Randomized, Controlled Study of Nanocrystalline Silver, Manuka Honey, and Conventional Dressing in Healing Diabetic Foot Ulcer. Evid-Based Complement Altern Med ECAM. 2017;2017:5294890.

37. Bergqvist K, Almhöjd U, Herrmann I, Eliasson B. The role of chloramines in treatment of diabetic foot ulcers: an exploratory multicentre randomised controlled trial. Clin Diabetes Endocrinol. 2016;2:6.

38. Siavash M, Shokri S, Haghighi S, Shahtalebi MA, Farajzadehgan Z. The efficacy of topical royal jelly on healing of diabetic foot ulcers: a double-blind placebo-controlled clinical trial. Int Wound J. 2015 Apr;12(2):137–42.

39. Kamaratos AV, Tzirogiannis KN, Iraklianou SA, Panoutsopoulos GI, Kanellos IE, Melidonis AI. Manuka honey-impregnated dressings in the treatment of neuropathic diabetic foot ulcers. Int Wound J. 2014 Jun;11(3):259–63.

40. Tian X., Yi L.-J., Ma L., Zhang L., Song G.-M., Wang Y. Effects of honey dressing for the treatment of DFUs: A systematic review. Int J Nurs Sci. 2014;1(2):224–31.

41. Mujica V, Orrego R, Fuentealba R, Leiva E, Zuniga-Hernandez J. Propolis as an Adjuvant in the Healing of Human Diabetic Foot Wounds Receiving Care in the Diagnostic and Treatment Centre from the Regional Hospital of Talca. J Diabetes Res. 2019;2019:2507578.

42. Afkhamizadeh M, Aboutorabi R, Ravari H, Fathi Najafi M, Ataei Azimi S, Javadian Langaroodi A, et al. Topical propolis improves wound healing in patients with diabetic foot ulcer: a randomized controlled trial. Nat Prod Res. 2018 Sep;32(17):2096–9.

43. Motley TA, Caporusso JM, Lange DL, Eichelkraut RA, Cargill DI, Dickerson JE. Clinical Outcomes for Diabetic Foot Ulcers Treated with Clostridial Collagenase Ointment or with a Product Containing Silver. Adv Wound Care. 2018 Oct 1;7(10):339–48.

44. Varga M, Sixta B, Bem R, Matia I, Jirkovska A, Adamec M. Application of gentamicin-collagen sponge shortened wound healing time after minor amputations in diabetic patients - a prospective, randomised trial. Arch Med Sci AMS. 2014 May 12;10(2):283–7.

45. Zhang L, Yin H, Lei X, Lau JNY, Yuan M, Wang X, et al. A Systematic Review and Meta-Analysis of Clinical Effectiveness and Safety of Hydrogel Dressings in the Management of Skin Wounds. Front Bioeng Biotechnol. 2019;7:342.

46. Gallelli G, Cione E, Serra R, Leo A, Citraro R, Matricardi P, et al. Nano-hydrogel embedded with quercetin and oleic acid as a new formulation in the treatment of diabetic foot ulcer: A pilot study. Int Wound J. 2020 Apr;17(2):485–90.

47. Edmonds M, Lázaro-Martínez JL, Alfayate-García JM, Martini J, Petit JM, Rayman G, et al. Sucrose octasulfate dressing versus control dressing in patients with neuroischaemic diabetic foot ulcers (Explorer): an international, multicentre, double-blind, randomised, controlled trial. Lancet Diabetes Endocrinol. 2018;6(3):186–96.

48. Tonaco LAB, Gomes FL, Velasquez-Melendez G, Lopes MTP, Salas CE. The Proteolytic Fraction from Latex of Vasconcellea cundinamarcensis (P1G10) Enhances Wound Healing of Diabetic Foot Ulcers: A Double-Blind Randomized Pilot Study. Adv Ther. 2018;35(4):494–502.

49. Saco M, Howe N, Nathoo R, Cherpelis B. Comparing the efficacies of alginate, foam, hydrocolloid, hydrofiber, and hydrogel dressings in the management of diabetic foot ulcers and venous leg ulcers: a systematic review and meta-analysis examining how to dress for success. Dermatol Online J. 2016 Aug 15;22(8).

50. Lee M, Han SH, Choi WJ, Chung KH, Lee JW. Hyaluronic acid dressing (Healoderm) in the treatment of diabetic foot ulcer: A prospective, randomized, placebo-controlled, single-center study. Wound Repair Regen Off Publ Wound Heal Soc Eur Tissue Repair Soc. 2016;24(3):581–8.

51. Grek CL, Prasad GM, Viswanathan V, Armstrong DG, Gourdie RG, Ghatnekar GS. Topical administration of a connexin43-based peptide augments healing of chronic neuropathic diabetic foot ulcers: A multicenter, randomized trial. Wound Repair Regen Off Publ Wound Heal Soc Eur Tissue Repair Soc. 2015 Apr;23(2):203–12.

52. Chen CP, Hung W, Lin SH. Effectiveness of hyaluronic acid for treating diabetic foot: a systematic review and meta-analysis. Dermatol Ther. 2014 Dec;27(6):331–6.

53. Dumville JC, O’Meara S, Deshpande S, Speak K. Alginate dressings for healing diabetic foot ulcers. Cochrane Database Syst Rev. 2013 Jun 25;(6):CD009110.

54. Voigt J, Driver VR. Hyaluronic acid derivatives and their healing effect on burns, epithelial surgical wounds, and chronic wounds: a systematic review and meta-analysis of randomized controlled trials. Wound Repair Regen Off Publ Wound Heal Soc Eur Tissue Repair Soc. 2012 Jun;20(3):317–31.

55. Dumville JC, Soares MO, O’Meara S, Cullum N. Systematic review and mixed treatment comparison: dressings to heal diabetic foot ulcers. Diabetologia. 2012 Jul;55(7):1902–10.

56. Dumville JC, Deshpande S, O’Meara S, Speak K. Hydrocolloid dressings for healing diabetic foot ulcers. Cochrane Database Syst Rev. 2013 Aug 6;(8):CD009099.

57. Dumville JC, O’Meara S, Deshpande S, Speak K. Hydrogel dressings for healing diabetic foot ulcers. Cochrane Database Syst Rev. 2013 Jul 12;(7):CD009101.

58. Dumville JC, Deshpande S, O’Meara S, Speak K. Foam dressings for healing diabetic foot ulcers. Cochrane Database Syst Rev. 2013 Jun 6;(6):CD009111.

59. Gwak HC, Han SH, Lee J, Park S, Sung KS, Kim HJ, et al. Efficacy of a povidone-iodine foam dressing (Betafoam) on diabetic foot ulcer. Int Wound J. 2020 Feb;17(1):91–9.

60. Blume P, Driver VR, Tallis AJ, Kirsner RS, Kroeker R, Payne WG, et al. Formulated collagen gel accelerates healing rate immediately after application in patients with diabetic neuropathic foot ulcers. Wound Repair Regen Off Publ Wound Heal Soc Eur Tissue Repair Soc. 2011 Jun;19(3):302–8.

61. Shaw J, Hughes CM, Lagan KM, Stevenson MR, Irwin CR, Bell PM. The effect of topical phenytoin on healing in diabetic foot ulcers: a randomized controlled trial. Diabet Med J Br Diabet Assoc. 2011 Oct;28(10):1154–7.

62. Bhittani MK, Rehman M, Altaf HN, Altaf OS. Effectiveness of Topical Insulin Dressings in Management of Diabetic Foot Ulcers. World J Surg. 2019 Dec 9;

63. Meimeti E, Tentolouris N, Manes C, Loupa C, Provatopoulou X, Mostratos D, et al. Ointments containing Ceratothoa oestroides extract: Evaluation of their healing potential in the treatment of diabetic foot ulcers. Wound Repair Regen Off Publ Wound Heal Soc Eur Tissue Repair Soc. 2019 Oct 16;

64. Motawea A, Abd El-Gawad AEGH, Borg T, Motawea M, Tarshoby M. The impact of topical phenytoin loaded nanostructured lipid carriers in diabetic foot ulceration. Foot Edinb Scotl. 2019 Sep;40:14–21.

65. Delgado-Enciso I, Madrigal-Perez VM, Lara-Esqueda A, Diaz-Sanchez MG, Guzman-Esquivel J, Rosas-Vizcaino LE, et al. Topical 5% potassium permanganate solution accelerates the healing process in chronic diabetic foot ulcers. Biomed Rep. 2018 Feb;8(2):156–9.

66. Prabhu R, Ravi C, Pai S, Rodrigues G. The efficacy of topical phenytoin in the healing of diabetic foot ulcers: a randomized double-blinded trial. Int J Diabetes Dev Ctries. 2017 Mar;37(1):46–9.

67. Ahmed A., Ahmed M.I. A comparison of efficacy of topical use of phenytoin and vaseline gauze dressing with vaseline gauze dressing alone in healing of diabetic foot ulcers. J Postgrad Med Inst. 2014;28(3):297–302.

68. Gottrup F, Cullen BM, Karlsmark T, Bischoff-Mikkelsen M, Nisbet L, Gibson MC. Randomized controlled trial on collagen/oxidized regenerated cellulose/silver treatment. Wound Repair Regen Off Publ Wound Heal Soc Eur Tissue Repair Soc. 2013 Apr;21(2):216–25.

69. Solway DR, Clark WA, Levinson DJ. A parallel open-label trial to evaluate microbial cellulose wound dressing in the treatment of diabetic foot ulcers. Int Wound J. 2011 Feb;8(1):69–73.

70. Franck Maunoury, Anaïs Oury, Sophie Fortin. Cost-effectiveness of TLC-NOSF dressings versus neutral dressings for the treatment of diabetic foot ulcers in France (NCT01717183). https://clinicaltrials.gov/show/NCT01717183 [Internet]. 2021; Available from: https://www.cochranelibrary.com/central/doi/10.1002/central/CN-02024806/full

71. Cutting KF. The cost-effectiveness of a novel soluble beta-glucan gel. J Wound Care. 2017 May 2;26(5):228–34.

72. Bilyayeva OO, Neshta VV, Golub AA, Sams-Dodd F. Comparative Clinical Study of the Wound Healing Effects of a Novel Micropore Particle Technology: Effects on Wounds, Venous Leg Ulcers, and Diabetic Foot Ulcers. Wounds Compend Clin Res Pract. 2017;29(8):1–9.

73. Patil V, Patil R, Kariholu PL, Patil LS, Shahapur P. Topical Phenytoin Application in Grade I and II Diabetic Foot Ulcers: A Prospective Study. J Clin Diagn Res JCDR. 2013 Oct;7(10):2238–40.

74. Alvaro-Afonso FJ, Garcia-Alvarez Y, Lazaro-Martinez JL, Kakagia D, Papanas N. Advances in Dermoepidermal Skin Substitutes for Diabetic Foot Ulcers. Curr Vasc Pharmacol. 2020;18(2):182–92.

75. Campitiello F, Mancone M, Della Corte A, Guerniero R, Canonico S. To evaluate the efficacy of an acellular Flowable matrix in comparison with a wet dressing for the treatment of patients with diabetic foot ulcers: a randomized clinical trial. Updat Surg. 2017 Dec;69(4):523–9.

76. Guo X, Mu D, Gao F. Efficacy and safety of acellular dermal matrix in diabetic foot ulcer treatment: A systematic review and meta-analysis. Int J Surg Lond Engl. 2017 Apr;40:1–7.

77. Tchero H, Herlin C, Bekara F, Kangambega P, Sergiu F, Teot L. Failure rates of artificial dermis products in treatment of diabetic foot ulcer: A systematic review and network meta-analysis. Wound Repair Regen Off Publ Wound Heal Soc Eur Tissue Repair Soc. 2017;25(4):691–6.

78. Cazzell S, Vayser D, Pham H, Walters J, Reyzelman A, Samsell B, et al. A randomized clinical trial of a human acellular dermal matrix demonstrated superior healing rates for chronic diabetic foot ulcers over conventional care and an active acellular dermal matrix comparator. Wound Repair Regen Off Publ Wound Heal Soc Eur Tissue Repair Soc. 2017;25(3):483–97.

79. Chandler LA, Alvarez OM, Blume PA, Kim PJ, Kirsner RS, Lantis JC, et al. Wound Conforming Matrix Containing Purified Homogenate of Dermal Collagen Promotes Healing of Diabetic Neuropathic Foot Ulcers: Comparative Analysis Versus Standard of Care. Adv Wound Care. 2020 Feb 1;9(2):61–7.

80. Santema TBK, Poyck PPC, Ubbink DT. Systematic review and meta-analysis of skin substitutes in the treatment of diabetic foot ulcers: Highlights of a Cochrane systematic review. Wound Repair Regen Off Publ Wound Heal Soc Eur Tissue Repair Soc. 2016;24(4):737–44.

81. Hu Z, Zhu J, Cao X, Chen C, Li S, Guo D, et al. Composite Skin Grafting with Human Acellular Dermal Matrix Scaffold for Treatment of Diabetic Foot Ulcers: A Randomized Controlled Trial. J Am Coll Surg. 2016;222(6):1171–9.

82. Huang W, Chen Y, Wang N, Yin G, Wei C, Xu W. The Efficacy and Safety of Acellular Matrix Therapy for Diabetic Foot Ulcers: A Meta-Analysis of Randomized Clinical Trials. J Diabetes Res. 2020;2020:6245758.

83. Park KH, Kwon JB, Park JH, Shin JC, Han SH, Lee JW. Collagen dressing in the treatment of diabetic foot ulcer: A prospective, randomized, placebo-controlled, single-center study. Diabetes Res Clin Pract. 2019 Oct;156:107861.

84. Alvarez OM, Smith T, Gilbert TW, Onumah NJ, Wendelken ME, Parker R, et al. Diabetic Foot Ulcers Treated With Porcine Urinary Bladder Extracellular Matrix and Total Contact Cast: Interim Analysis of a Randomized, Controlled Trial. Wounds Compend Clin Res Pract. 2017;29(5):140–6.

85. Walters J, Cazzell S, Pham H, Vayser D, Reyzelman A. Healing Rates in a Multicenter Assessment of a Sterile, Room Temperature, Acellular Dermal Matrix Versus Conventional Care Wound Management and an Active Comparator in the Treatment of Full-Thickness Diabetic Foot Ulcers. Eplasty. 2016;16:e10.

86. Driver V.R., Lavery L.A., Reyzelman A.M., Dutra T.G., Dove C.R., Kotsis S.V., et al. A clinical trial of Integra Template for diabetic foot ulcer treatment. Wound Repair Regen. 2015;23(6):891–900.

87. Şevki Çetinkalp, Evren Homan Gökçe, IlgınYıldırım Şimşir, Sakine Tuncay Tanrıverdi. Comparative Evaluation of Clinical Efficacy and Safety of Collagen Laminin–Based Dermal Matrix Combined With Resveratrol Microparticles (Dermalix) and Standard Wound Care for Diabetic Foot Ulcers - NCT03136822. https://clinicaltrials.gov/show/NCT03136822 [Internet]. 2020; Available from: https://www.cochranelibrary.com/central/doi/10.1002/central/CN-01580859/full

88. Serena TE, Yaakov R, Moore S, Cole W, Coe S, Snyder R, et al. A randomized controlled clinical trial of a hypothermically stored amniotic membrane for use in diabetic foot ulcers. J Comp Eff Res. 2020 Jan;9(1):23–34.

89. Luck J, Rodi T, Geierlehner A, Mosahebi A. Allogeneic Skin Substitutes Versus Human Placental Membrane Products in the Management of Diabetic Foot Ulcers: A Narrative Comparative Evaluation of the Literature. Int J Low Extrem Wounds. 2019 Mar;18(1):10–22.

90. Haugh AM, Witt JG, Hauch A, Darden M, Parker G, Ellsworth WA, et al. Amnion Membrane in Diabetic Foot Wounds: A Meta-analysis. Plast Reconstr Surg Glob Open. 2017 Apr;5(4):e1302.

91. Mohajeri-Tehrani MR, Variji Z, Mohseni S, Firuz A, Annabestani Z, Zartab H, et al. Comparison of a Bioimplant Dressing With a Wet Dressing for the Treatment of Diabetic Foot Ulcers: A Randomized, Controlled Clinical Trial. Wounds Compend Clin Res Pract. 2016 Jul;28(7):248–54.

92. Snyder RJ, Shimozaki K, Tallis A, Kerzner M, Reyzelman A, Lintzeris D, et al. A Prospective, Randomized, Multicenter, Controlled Evaluation of the Use of Dehydrated Amniotic Membrane Allograft Compared to Standard of Care for the Closure of Chronic Diabetic Foot Ulcer. Wounds Compend Clin Res Pract. 2016 Mar;28(3):70–7.

93. Lavery LA, Fulmer J, Shebetka KA, Regulski M, Vayser D, Fried D, et al. The efficacy and safety of Grafix(®) for the treatment of chronic diabetic foot ulcers: results of a multi-centre, controlled, randomised, blinded, clinical trial. Int Wound J. 2014 Oct;11(5):554–60.

94. Zelen CM, Serena TE, Snyder RJ. A prospective, randomised comparative study of weekly versus biweekly application of dehydrated human amnion/chorion membrane allograft in the management of diabetic foot ulcers. Int Wound J. 2014 Apr;11(2):122–8.

95. Greer N., Foman N.A., MacDonald R., Dorrian J., Fitzgerald P., Rutks I., et al. Advanced wound care therapies for nonhealing diabetic, venous, and arterial ulcers: A systematic review. Ann Intern Med. 2013;159(8):532–42.

96. You HJ, Han SK, Rhie JW. Randomised controlled clinical trial for autologous fibroblast-hyaluronic acid complex in treating diabetic foot ulcers. J Wound Care. 2014 Nov;23(11):521–2, 524, 526–30.

97. Su YN, Zhao DY, Li YH, Yu TQ, Sun H, Wu XY, et al. Human amniotic membrane allograft, a novel treatment for chronic diabetic foot ulcers: A systematic review and meta-analysis of randomised controlled trials. Int Wound J. 2020 Mar 2;

98. Gordon AJ, Alfonso AR, Nicholson J, Chiu ES. Evidence for Healing Diabetic Foot Ulcers With Biologic Skin Substitutes: A Systematic Review and Meta-Analysis. Ann Plast Surg. 2019 Oct;83(4S Suppl 1):S31–44.

99. DiDomenico LA, Orgill DP, Galiano RD, Serena TE, Carter MJ, Kaufman JP, et al. Use of an aseptically processed, dehydrated human amnion and chorion membrane improves likelihood and rate of healing in chronic diabetic foot ulcers: A prospective, randomised, multi-centre clinical trial in 80 patients. Int Wound J. 2018 Dec;15(6):950–7.

100. Zelen CM, Orgill DP, Serena TE, Galiano RE, Carter MJ, DiDomenico LA, et al. An aseptically processed, acellular, reticular, allogenic human dermis improves healing in diabetic foot ulcers: A prospective, randomised, controlled, multicentre follow-up trial. Int Wound J. 2018 Oct;15(5):731–9.

101. Zelen CM, Serena TE, Denoziere G, Fetterolf DE. A prospective randomised comparative parallel study of amniotic membrane wound graft in the management of diabetic foot ulcers. Int Wound J. 2013 Oct;10(5):502–7.

102. Glat P, Orgill DP, Galiano R, Armstrong D, Serena T, DiDomenico LA, et al. Placental Membrane Provides Improved Healing Efficacy and Lower Cost Versus a Tissue-Engineered Human Skin in the Treatment of Diabetic Foot Ulcerations. Plast Reconstr Surg Glob Open. 2019 Aug;7(8):e2371.

103. Tettelbach W, Cazzell S, Sigal F, Caporusso JM, Agnew PS, Hanft J, et al. A multicentre prospective randomised controlled comparative parallel study of dehydrated human umbilical cord (EpiCord) allograft for the treatment of diabetic foot ulcers. Int Wound J. 2019 Feb;16(1):122–30.

104. Zelen CM, Serena TE, Gould L, Le L, Carter MJ, Keller J, et al. Treatment of chronic diabetic lower extremity ulcers with advanced therapies: a prospective, randomised, controlled, multi-centre comparative study examining clinical efficacy and cost. Int Wound J. 2016 Apr;13(2):272–82.

105. Cazzell SM, Lange DL, Dickerson JE, Slade HB. The Management of Diabetic Foot Ulcers with Porcine Small Intestine Submucosa Tri-Layer Matrix: A Randomized Controlled Trial. Adv Wound Care. 2015 Dec 1;4(12):711–8.

106. Tettelbach W., Cazzell S., Reyzelman A.M., Sigal F., Caporusso J.M., Agnew P.S. A confirmatory study on the efficacy of dehydrated human amnion/chorion membrane dHACM allograft in the management of diabetic foot ulcers: A prospective, multicentre, randomised, controlled study of 110 patients from 14 wound clinics. Int Wound J. 2018;16(1):19–29.

107. Li X, Xu G, Chen J. Tissue engineered skin for diabetic foot ulcers: a meta-analysis. Int J Clin Exp Med. 2015;8(10):18191–6.

108. Lu D, Jiang Y, Deng W, Zhang Y, Liang Z, Wu Q, et al. Long-Term Outcomes of BMMSC Compared with BMMNC for Treatment of Critical Limb Ischemia and Foot Ulcer in Patients with Diabetes. Cell Transplant. 2019 May;28(5):645–52.

109. You HJ, Han SK, Lee JW, Chang H. Treatment of diabetic foot ulcers using cultured allogeneic keratinocytes--a pilot study. Wound Repair Regen Off Publ Wound Heal Soc Eur Tissue Repair Soc. 2012 Aug;20(4):491–9.

110. Ananian CE, Dhillon YS, Van Gils CC, Lindsey DC, Otto RJ, Dove CR, et al. A multicenter, randomized, single-blind trial comparing the efficacy of viable cryopreserved placental membrane to human fibroblast-derived dermal substitute for the treatment of chronic diabetic foot ulcers. Wound Repair Regen Off Publ Wound Heal Soc Eur Tissue Repair Soc. 2018;26(3):274–83.

111. Laurent I, Astère M, Wang KR, Cheng QF, Li QF. Efficacy and Time Sensitivity of Amniotic Membrane treatment in Patients with Diabetic Foot Ulcers: A Systematic Review and Meta-analysis. Diabetes Ther Res Treat Educ Diabetes Relat Disord. 2017 Oct;8(5):967–79.

112. Guest JF, Weidlich D, Singh H, La Fontaine J, Garrett A, Abularrage CJ, et al. Cost-effectiveness of using adjunctive porcine small intestine submucosa tri-layer matrix compared with standard care in managing diabetic foot ulcers in the US. J Wound Care. 2017 02;26(Sup1):S12–24.

113. Langer A, Rogowski W, Heybeck TV. Systematic Review of Economic Evaluations of Human Cell-Derived Wound C are Products for the Treatment of Venous Leg and Diabetic Foot Ulcers. Foot Ankle Q-- Semin J [Internet]. 2016;27(9). Available from: http://search.ebscohost.com/login.aspx?direct=true&db=cin20&AN=114503321&site=ehost-live&scope=site

114. Rice JB, Desai U, Ristovska L, Cummings AKG, Birnbaum HG, Skornicki M, et al. Economic outcomes among Medicare patients receiving bioengineered cellular technologies for treatment of diabetic foot ulcers. J Med Econ. 2015;18(8):586–95.

115. Lonardi R, Leone N, Gennai S, Trevisi Borsari G, Covic T, Silingardi R. Autologous micro-fragmented adipose tissue for the treatment of diabetic foot minor amputations: a randomized controlled single-center clinical trial (MiFrAADiF). Stem Cell Res Ther. 2019 Jul 29;10(1):223.

116. Li L, Chen D, Wang C, Yuan N, Wang Y, He L, et al. Autologous platelet-rich gel for treatment of diabetic chronic refractory cutaneous ulcers: A prospective, randomized clinical trial. Wound Repair Regen Off Publ Wound Heal Soc Eur Tissue Repair Soc. 2015 Aug;23(4):495–505.

117. Game F, Jeffcoate W, Tarnow L, Jacobsen JL, Whitham DJ, Harrison EF, et al. LeucoPatch system for the management of hard-to-heal diabetic foot ulcers in the UK, Denmark, and Sweden: an observer-masked, randomised controlled trial. Lancet Diabetes Endocrinol. 2018;6(11):870–8.

118. Uccioli L, Giurato L, Ruotolo V, Ciavarella A, Grimaldi MS, Piaggesi A, et al. Two-step autologous grafting using HYAFF scaffolds in treating difficult diabetic foot ulcers: results of a multicenter, randomized controlled clinical trial with long-term follow-up. Int J Low Extrem Wounds. 2011 Jun;10(2):80–5.

119. Zhang Y, Deng H, Tang Z. Efficacy of Cellular Therapy for Diabetic Foot Ulcer: A Meta-Analysis of Randomized Controlled Clinical Trials. Cell Transplant. 2017;26(12):1931–9.

120. Dubsky M, Jirkovska A, Bem R, Fejfarova V, Pagacova L, Sixta B, et al. Both autologous bone marrow mononuclear cell and peripheral blood progenitor cell therapies similarly improve ischaemia in patients with diabetic foot in comparison with control treatment. Diabetes Metab Res Rev. 2013 Jul;29(5):369–76.

121. Moon KC, Suh HS, Kim KB, Han SK, Young KW, Lee JW, et al. Potential of Allogeneic Adipose-Derived Stem Cell-Hydrogel Complex for Treating Diabetic Foot Ulcers. Diabetes. 2019;68(4):837–46.

122. Shu X, Shu S, Tang S, Yang L, Liu D, Li K, et al. Efficiency of stem cell based therapy in the treatment of diabetic foot ulcer: a meta-analysis. Endocr J. 2018 Apr 26;65(4):403–13.

123. Guo J, Dardik A, Fang K, Huang R, Gu Y. Meta-analysis on the treatment of diabetic foot ulcers with autologous stem cells. Stem Cell Res Ther. 2017 16;8(1):228.

124. Kirana S, Stratmann B, Prante C, Prohaska W, Koerperich H, Lammers D, et al. Autologous stem cell therapy in the treatment of limb ischaemia induced chronic tissue ulcers of diabetic foot patients. Int J Clin Pract. 2012 Apr;66(4):384–93.

125. Golledge J, Singh TP. Systematic review and meta-analysis of clinical trials examining the effect of hyperbaric oxygen therapy in people with diabetes-related lower limb ulcers. Diabet Med J Br Diabet Assoc. 2019 Jul;36(7):813–26.

126. Salama SE, Eldeeb AE, Elbarbary AH, Abdelghany SE. Adjuvant Hyperbaric Oxygen Therapy Enhances Healing of Nonischemic Diabetic Foot Ulcers Compared With Standard Wound Care Alone. Int J Low Extrem Wounds. 2019 Mar;18(1):75–80.

127. Chen CY, Wu RW, Hsu MC, Hsieh CJ, Chou MC. Adjunctive Hyperbaric Oxygen Therapy for Healing of Chronic Diabetic Foot Ulcers: A Randomized Controlled Trial. J Wound Ostomy Cont Nurs Off Publ Wound Ostomy Cont Nurses Soc. 2017 Dec;44(6):536–45.

128. Health Quality Ontario. Hyperbaric Oxygen Therapy for the Treatment of Diabetic Foot Ulcers: A Health Technology Assessment. Ont Health Technol Assess Ser. 2017;17(5):1–142.

129. Elraiyah T, Tsapas A, Prutsky G, Domecq JP, Hasan R, Firwana B, et al. A systematic review and meta-analysis of adjunctive therapies in diabetic foot ulcers. J Vasc Surg. 2016 Feb;63(2 Suppl):46S-58S.e1-2.

130. Liu R, Li L, Yang M, Boden G, Yang G. Systematic review of the effectiveness of hyperbaric oxygenation therapy in the management of chronic diabetic foot ulcers. Mayo Clin Proc. 2013 Feb;88(2):166–75.

131. O’Reilly D, Pasricha A, Campbell K, Burke N, Assasi N, Bowen JM, et al. Hyperbaric oxygen therapy for diabetic ulcers: systematic review and meta-analysis. Int J Technol Assess Health Care. 2013 Jul;29(3):269–81.

132. Lalieu RC, Brouwer RJ, Ubbink DT, Hoencamp R, Bol Raap R, van Hulst RA. Hyperbaric oxygen therapy for nonischemic diabetic ulcers: A systematic review. Wound Repair Regen Off Publ Wound Heal Soc Eur Tissue Repair Soc. 2019 Oct 31;

133. Santema KTB, Stoekenbroek RM, Koelemay MJW, Reekers JA, van Dortmont LMC, Oomen A, et al. Hyperbaric Oxygen Therapy in the Treatment of Ischemic Lower- Extremity Ulcers in Patients With Diabetes: Results of the DAMO2CLES Multicenter Randomized Clinical Trial. Diabetes Care. 2018;41(1):112–9.

134. Zhao D, Luo S, Xu W, Hu J, Lin S, Wang N. Efficacy and Safety of Hyperbaric Oxygen Therapy Used in Patients With Diabetic Foot: A Meta-analysis of Randomized Clinical Trials. Clin Ther. 2017 Oct;39(10):2088-2094.e2.

135. Kranke P, Bennett MH, Martyn-St James M, Schnabel A, Debus SE, Weibel S. Hyperbaric oxygen therapy for chronic wounds. Cochrane Database Syst Rev. 2015 Jun 24;(6):CD004123.

136. Margolis DJ, Gupta J, Hoffstad O, Papdopoulos M, Glick HA, Thom SR, et al. Lack of effectiveness of hyperbaric oxygen therapy for the treatment of diabetic foot ulcer and the prevention of amputation: a cohort study. Diabetes Care. 2013 Jul;36(7):1961–6.

137. Perren S, Gatt A, Papanas N, Formosa C. Hyperbaric Oxygen Therapy in Ischaemic Foot Ulcers in Type 2 Diabetes: A Clinical Trial. Open Cardiovasc Med J. 2018;12:80–5.

138. MOHD YAZID B, AYESYAH A, NURHANANI AB, MOHD ROHAIZAT H. The Physiological, Biochemical and Quality of Life Changes in Chronic Diabetic Foot Ulcer after Hyperbaric Oxygen Therapy. Med Health Univ Kebangs Malays. 2017 Jul;12(2):210–9.

139. Fedorko L, Bowen JM, Jones W, Oreopoulos G, Goeree R, Hopkins RB, et al. Hyperbaric Oxygen Therapy Does Not Reduce Indications for Amputation in Patients With Diabetes With Nonhealing Ulcers of the Lower Limb: A Prospective, Double-Blind, Randomized Controlled Clinical Trial. Diabetes Care. 2016 Mar;39(3):392–9.

140. Löndahl M, Landin-Olsson M, Katzman P. Hyperbaric oxygen therapy improves health-related quality of life in patients with diabetes and chronic foot ulcer. Diabet Med J Br Diabet Assoc. 2011 Feb;28(2):186–90.

141. Li G, Hopkins RB, Levine MAH, Jin X, Bowen JM, Thabane L, et al. Relationship between hyperbaric oxygen therapy and quality of life in participants with chronic diabetic foot ulcers: data from a randomized controlled trial. Acta Diabetol. 2017 Sep;54(9):823–31.

142. Eggert JV, Worth ER, Van Gils CC. Cost and mortality data of a regional limb salvage and hyperbaric medicine program for Wagner Grade 3 or 4 diabetic foot ulcers. Undersea Hyperb Med J Undersea Hyperb Med Soc Inc. 2016 Feb;43(1):1–8.

143. Hamed S, Ullmann, Belokopytov M. Topical Erythropoietin Accelerates Wound Closure in Patients with Diabetic Foot Ulcers: A Prospective, Multicenter, Single-Blind, Randomized, Controlled Trial - NCT02361931. https://clinicaltrials.gov/show/NCT02361931 [Internet]. 2021; Available from: https://www.cochranelibrary.com/central/doi/10.1002/central/CN-02044267/full

144. Park KH, Han SH, Hong JP, Han SK, Lee DH, Kim BS, et al. Topical epidermal growth factor spray for the treatment of chronic diabetic foot ulcers: A phase III multicenter, double-blind, randomized, placebo-controlled trial. Diabetes Res Clin Pract. 2018 Aug;142:335–44.

145. Sridharan K, Sivaramakrishnan G. Growth factors for diabetic foot ulcers: mixed treatment comparison analysis of randomized clinical trials. Br J Clin Pharmacol. 2018;84(3):434–44.

146. Martinez-Zapata MJ, Martí-Carvajal AJ, Solà I, Expósito JA, Bolíbar I, Rodríguez L, et al. Autologous platelet-rich plasma for treating chronic wounds. Cochrane Database Syst Rev. 2016 May 25;(5):CD006899.

147. Yang S, Geng Z, Ma K, Sun X, Fu X. Efficacy of Topical Recombinant Human Epidermal Growth Factor for Treatment of Diabetic Foot Ulcer: A Systematic Review and Meta-Analysis. Int J Low Extrem Wounds. 2016 Jun;15(2):120–5.

148. Bui T, Bui Q, Németh D, Hegyi P, Szakács Z, Rumbus Z, et al. Epidermal Growth Factor is Effective in the Treatment of Diabetic Foot Ulcers: Meta-Analysis and Systematic Review. Int J Environ Res Public Health. 2019 Jul 19;16:2584.

149. Gomez-Villa R, Aguilar-Rebolledo F, Lozano-Platonoff A, Teran-Soto JM, Fabian-Victoriano MR, Kresch-Tronik NS, et al. Efficacy of intralesional recombinant human epidermal growth factor in diabetic foot ulcers in Mexican patients: a randomized double-blinded controlled trial. Wound Repair Regen Off Publ Wound Heal Soc Eur Tissue Repair Soc. 2014 Aug;22(4):497–503.

150. Elsaid A, El-Said M, Emile S, Youssef M, Khafagy W, Elshobaky A. Randomized Controlled Trial on Autologous Platelet-Rich Plasma Versus Saline Dressing in Treatment of Non-healing Diabetic Foot Ulcers. World J Surg. 2020 Apr;44(4):1294–301.

151. Gude W, Hagan D, Abood F, Clausen P. Aurix Gel Is an Effective Intervention for Chronic Diabetic Foot Ulcers: A Pragmatic Randomized Controlled Trial. Adv Skin Wound Care. 2019 Sep;32(9):416–26.

152. Li Y, Gao Y, Gao Y, Chen D, Wang C, Liu G, et al. Autologous platelet-rich gel treatment for diabetic chronic cutaneous ulcers: A meta-analysis of randomized controlled trials. J Diabetes. 2019 May;11(5):359–69.

153. Del Pino-Sedeño T, Trujillo-Martín MM, Andia I, Aragón-Sánchez J, Herrera-Ramos E, Iruzubieta Barragán FJ, et al. Platelet-rich plasma for the treatment of diabetic foot ulcers: A meta-analysis. Wound Repair Regen Off Publ Wound Heal Soc Eur Tissue Repair Soc. 2019;27(2):170–82.

154. Xia Y, Zhao J, Xie J, Lv Y, Cao DS. The Efficacy of Platelet-Rich Plasma Dressing for Chronic Nonhealing Ulcers: A Meta-Analysis of 15 Randomized Controlled Trials. Plast Reconstr Surg. 2019 Dec;144(6):1463–74.

155. Hu Z, Qu S, Zhang J, Cao X, Wang P, Huang S, et al. Efficacy and Safety of Platelet-Rich Plasma for Patients with Diabetic Ulcers: A Systematic Review and Meta-analysis. Adv Wound Care. 2019 Jul 1;8(7):298–308.

156. Picard F, Hersant B, Bosc R, Meningaud JP. The growing evidence for the use of platelet-rich plasma on diabetic chronic wounds: A review and a proposal for a new standard care. Wound Repair Regen Off Publ Wound Heal Soc Eur Tissue Repair Soc. 2015 Sep;23(5):638–43.

157. Martí-Carvajal AJ, Gluud C, Nicola S, Simancas-Racines D, Reveiz L, Oliva P, et al. Growth factors for treating diabetic foot ulcers. Cochrane Database Syst Rev. 2015 Oct 28;(10):CD008548.

158. Zhao X hong, Gu H feng, Xu Z rong, Zhang Q, Lv X ying, Zheng X jun, et al. Efficacy of topical recombinant human platelet-derived growth factor for treatment of diabetic lower-extremity ulcers: Systematic review and meta-analysis. Metabolism. 2014 Oct;63(10):1304–13.

159. Ding H, Fu XL, Miao WW, Mao XC, Zhan MQ, Chen HL. Efficacy of Autologous Platelet-Rich Gel for Diabetic Foot Wound Healing: A Meta-Analysis of 15 Randomized Controlled Trials. Adv Wound Care. 2019 May 1;8(5):195–207.

160. Shen Z, Zheng S, Chen G, Li D, Jiang Z, Li Y, et al. Efficacy and safety of platelet-rich plasma in treating cutaneous ulceration: A meta-analysis of randomized controlled trials. J Cosmet Dermatol. 2019 Apr;18(2):495–507.

161. Cruciani M, Lipsky BA, Mengoli C, de Lalla F. Granulocyte-colony stimulating factors as adjunctive therapy for diabetic foot infections. Cochrane Database Syst Rev. 2013 Aug 17;(8):CD006810.

162. Volpe P, Marcuccio D, Stilo G, Alberti A, Foti G, Volpe A, et al. Efficacy of cord blood platelet gel application for enhancing diabetic foot ulcer healing after lower limb revascularization. Semin Vasc Surg. 2017 Dec;30(4):106–12.

163. Hosseini S.E., Molavi B., Goodarzi A., Alizadeh A., Yousefzadeh A., Sodeifi N., et al. The efficacy of platelet gel derived from umbilical cord blood on diabetic foot ulcers: A double-blind randomized clinical trial. Wound Med [Internet]. 2020;28((Molavi B., molavibe@sina.tums.ac.ir) Sina Trauma and Surgery Research Center, Tehran University of Medical Sciences, Tehran, Iran). Available from: http://www.embase.com/search/results?subaction=viewrecord&from=export&id=L2004656584

164. Whitmont K, McKelvey K, Fulcher G, Reid I, March L, Xue M, et al. Treatment of chronic diabetic lower leg ulcers with activated protein C: a randomised placebo-controlled, double-blind pilot clinical trial. Int Wound J. 2015;12(4):422‐427.

165. Mohammadzadeh L, Samedanifard SH, Keshavarzi A, Alimoghaddam K, Larijani B, Ghavamzadeh A, et al. Therapeutic outcomes of transplanting autologous granulocyte colony-stimulating factor-mobilised peripheral mononuclear cells in diabetic patients with critical limb ischaemia. Exp Clin Endocrinol Diabetes Off J Ger Soc Endocrinol Ger Diabetes Assoc. 2013 Jan;121(1):48–53.

166. Xu J, Min D, Guo G, Liao X, Fu Z. Experimental study of epidermal growth factor and acidic fibroblast growth factor in the treatment of diabetic foot wounds. Exp Ther Med. 2018 Jun;15(6):5365–70.

167. Viswanathan V, Juttada U, Babu M. Efficacy of Recombinant Human Epidermal Growth Factor (Regen-D 150) in Healing Diabetic Foot Ulcers: A Hospital-Based Randomized Controlled Trial. Int J Low Extrem Wounds. 2019 Dec 26;1534734619892791.

168. Hirase T, Ruff E, Surani S, Ratnani I. Topical application of platelet-rich plasma for diabetic foot ulcers: A systematic review. World J Diabetes. 2018 Oct 15;9(10):172–9.

169. Ozturk A, Kucukardali Y, Tangi F, Erikci A, Uzun G, Bashekim C, et al. Therapeutical potential of autologous peripheral blood mononuclear cell transplantation in patients with type 2 diabetic critical limb ischemia. J Diabetes Complications. 2012 Feb;26(1):29–33.

170. Romero Prada M, Roa C, Alfonso P, Acero G, Huérfano L, Vivas-Consuelo D. Cost-effectiveness analysis of the human recombinant epidermal growth factor in the management of patients with diabetic foot ulcers. Diabet Foot Ankle. 2018;9(1):1480249.

171. Waycaster CR, Gilligan AM, Motley TA. Cost-Effectiveness of Becaplermin Gel on Diabetic Foot Ulcer HealingChanges in Wound Surface Area. J Am Podiatr Med Assoc. 2016 Jul;106(4):273–82.

172. Linertová R., Del Pino Sedeño T., García-Pérez L., Aragón-Sánchez J., Kaiser-Girardot S., Trujillo-Martín M., et al. PLATELET-RICH PLASMA IN DIABETIC FOOT ULCERS: COST-EFFECTIVENESS ANALYSIS FOR SPAIN. Value Health. 2018;21((Linertová R.) Fundación Canaria de Investigación Sanitaria (FUNCANIS), Santa Cruz de Tenerife, Spain):S130.

173. Gilligan AM, Waycaster CR, Motley TA. Cost-effectiveness of becaplermin gel on wound healing of diabetic foot ulcers. Wound Repair Regen Off Publ Wound Heal Soc Eur Tissue Repair Soc. 2015 Jun;23(3):353–60.

174. Tesar T, Szilberhorn L, Nemeth B, Nagy B, Wawruch M, Kalo Z. Cost-Utility Analysis of Heberprot-P as an Add-on Therapy to Good Wound Care for Patients in Slovakia with Advanced Diabetic Foot Ulcer. Front Pharmacol. 2017;8:946.

175. Mohseni S, Aalaa M, Atlasi R, Mohajeri Tehrani MR, Sanjari M, Amini MR. The effectiveness of negative pressure wound therapy as a novel management of diabetic foot ulcers: an overview of systematic reviews. J Diabetes Metab Disord. 2019 Dec;18(2):625–41.

176. Liu Z, Dumville JC, Hinchliffe RJ, Cullum N, Game F, Stubbs N, et al. Negative pressure wound therapy for treating foot wounds in people with diabetes mellitus. Cochrane Database Syst Rev. 2018 17;10:CD010318.

177. Liu S, He CZ, Cai YT, Xing QP, Guo YZ, Chen ZL, et al. Evaluation of negative-pressure wound therapy for patients with diabetic foot ulcers: systematic review and meta-analysis. Ther Clin Risk Manag. 2017;13:533–44.

178. Sihag B, Chahar C, Sharma J, Agrawal R. Trial of vacuum-assisted closure in patients with chronic nonhealing leg ulcers in type 2 diabetes mellitus. Diabetes. 2016;65:A555‐.

179. Wang R, Feng Y, Di B. Comparisons of negative pressure wound therapy and ultrasonic debridement for diabetic foot ulcers: a network meta-analysis. Int J Clin Exp Med. 2015;8(8):12548–56.

180. Zhang J, Hu ZC, Chen D, Guo D, Zhu JY, Tang B. Effectiveness and safety of negative-pressure wound therapy for diabetic foot ulcers: a meta-analysis. Plast Reconstr Surg. 2014 Jul;134(1):141–51.

181. Guffanti A. Negative pressure wound therapy in the treatment of diabetic foot ulcers: a systematic review of the literature. J Wound Ostomy Cont Nurs Off Publ Wound Ostomy Cont Nurses Soc. 2014 Jun;41(3):233–7.

182. Seidel D, Storck M, Lawall H, Wozniak G. Negative pressure wound therapy compared with standard moist wound care on diabetic foot ulcers in real-life clinical practice: results of the German DiaFu-RCT - NCT01480362. https://clinicaltrials.gov/show/NCT01480362 [Internet]. 2020; Available from: https://www.cochranelibrary.com/central/doi/10.1002/central/CN-01534125/full

183. Huang Q, Wang JT, Gu HC, Cao G, Cao JC. Comparison of Vacuum Sealing Drainage and Traditional Therapy for Treatment of Diabetic Foot Ulcers: A Meta-Analysis. J Foot Ankle Surg Off Publ Am Coll Foot Ankle Surg. 2019 Sep;58(5):954–8.

184. Sajid MT, Mustafa Q ul A, Shaheen N, Hussain SM, Shukr I, Ahmed M. Comparison of Negative Pressure Wound Therapy Using Vacuum-Assisted Closure with Advanced Moist Wound Therapy in the Treatment of Diabetic Foot Ulcers. J Coll Physicians Surg--Pak JCPSP. 2015 Nov;25(11):789–93.

185. Ravari H, Modaghegh MHS, Kazemzadeh GH, Johari HG, Vatanchi AM, Sangaki A, et al. Comparision of vacuum-asisted closure and moist wound dressing in the treatment of diabetic foot ulcers. J Cutan Aesthetic Surg. 2013 Jan;6(1):17–20.

186. Nain PS, Uppal SK, Garg R, Bajaj K, Garg S. Role of negative pressure wound therapy in healing of diabetic foot ulcers. J Surg Tech Case Rep. 2011 Jan;3(1):17–22.

187. Rys P, Borys S, Hohendorff J, Zapala A, Witek P, Monica M, et al. NPWT in diabetic foot wounds-a systematic review and meta-analysis of observational studies. Endocrine. 2020 Jan 9;

188. Wynn M, Freeman S. The efficacy of negative pressure wound therapy for diabetic foot ulcers: A systematised review. J Tissue Viability. 2019 Aug;28(3):152–60.

189. Karatepe O, Eken I, Acet E, Unal O, Mert M, Koc B, et al. Vacuum assisted closure improves the quality of life in patients with diabetic foot. Acta Chir Belg. 2011 Oct;111(5):298–302.

190. Driver V.R., Eckert K.A., Carter M.J., French M.A. Cost-effectiveness of negative pressure wound therapy in patients with many comorbidities and severe wounds of various etiology. Wound Repair Regen. 2016;24(6):1041–58.

191. Driver VR, Blume PA. Evaluation of wound care and health-care use costs in patients with diabetic foot ulcers treated with negative pressure wound therapy versus advanced moist wound therapy. J Am Podiatr Med Assoc. 2014 Mar;104(2):147–53.

192. Hutton DW, Sheehan P. Comparative effectiveness of the SNaP^TM^ Wound Care System. Int Wound J. 2011 Apr;8(2):196–205.

193. Whitehead SJ, Forest-Bendien VL, Richard JL, Halimi S, Van GH, Trueman P. Economic evaluation of Vacuum Assisted Closure® Therapy for the treatment of diabetic foot ulcers in France. Int Wound J. 2011 Feb;8(1):22–32.

194. Li S, Wang C, Wang B, Liu L, Tang L, Liu D, et al. Efficacy of low-level light therapy for treatment of diabetic foot ulcer: A systematic review and meta-analysis of randomized controlled trials. Diabetes Res Clin Pract. 2018 Sep;143:215–24.

195. Tchanque-Fossuo CN, Ho D, Dahle SE, Koo E, Li CS, Isseroff RR, et al. A systematic review of low-level light therapy for treatment of diabetic foot ulcer. Wound Repair Regen Off Publ Wound Heal Soc Eur Tissue Repair Soc. 2016;24(2):418–26.

196. Wang HT, Yuan JQ, Zhang B, Dong ML, Mao C, Hu D. Phototherapy for treating foot ulcers in people with diabetes. Cochrane Database Syst Rev. 2017 28;6:CD011979.

197. Haze A, Gavish L, Elishoov O, Shorka D. Treatment of diabetic foot ulcers in a frail population with severe co-morbidities using at-home photobiomodulation laser therapy: a double-blind, randomized, sham-controlled pilot clinical study - NCT01493895. https://clinicaltrials.gov/show/NCT01493895 [Internet]. 2021; Available from: https://www.cochranelibrary.com/central/doi/10.1002/central/CN-01534493/full

198. Ortíz M, Villabona E, Lemos D, Castellanos R. Effects of low level laser therapy and high voltage stimulation on diabetic wound healing. Rev Univ Ind Santander Salud. 2014;46(2):107‐117.

199. Vitoriano NAM, Mont’Alverne DGB, Martins MIS, Silva PS, Martins CA, Teixeira HD, et al. Comparative study on laser and LED influence on tissue repair and improvement of neuropathic symptoms during the treatment of diabetic ulcers. Lasers Med Sci. 2019 Sep;34(7):1365–71.

200. Alayat MS, El-Sodany AM, Ebid AA, Shousha TM, Abdelgalil AA, Alhasan H, et al. Efficacy of high intensity laser therapy in the management of foot ulcers: a systematic review. J Phys Ther Sci. 2018 Oct;30(10):1341–5.

201. Tantawy SA, Abdelbasset WK, Kamel DM, Alrawaili SM. A randomized controlled trial comparing helium-neon laser therapy and infrared laser therapy in patients with diabetic foot ulcer. Lasers Med Sci. 2018 Dec;33(9):1901–6.

202. de Alencar Fonseca Santos J, Campelo MBD, de Oliveira RA, Nicolau RA, Rezende VEA, Arisawa EÂL. Effects of Low-Power Light Therapy on the Tissue Repair Process of Chronic Wounds in Diabetic Feet. Photomed Laser Surg. 2018 Jun;36(6):298–304.

203. Carrinho PM, Andreani DIK, Morete V de A, Iseri S, Navarro RS, Villaverde AB. A Study on the Macroscopic Morphometry of the Lesion Area on Diabetic Ulcers in Humans Treated with Photodynamic Therapy Using Two Methods of Measurement. Photomed Laser Surg. 2018 Jan;36(1):44–50.

204. Srilestari A., Nareswari I., Simadibrata C., Tarigan T.J.E. Effectiveness of combined laser-puncture and conventional wound care to accelerate diabetic foot ulcer healing. Med J Indones. 2017;26(1):26–34.

205. Mathur RK, Sahu K, Saraf S, Patheja P, Khan F, Gupta PK. Low-level laser therapy as an adjunct to conventional therapy in the treatment of diabetic foot ulcers. Lasers Med Sci. 2017 Feb;32(2):275–82.

206. Carvalho AFM de, Feitosa MCP, Coelho NPM de F, Rebêlo VCN, Castro JG de, Sousa PRG de, et al. Low-level laser therapy and Calendula officinalis in repairing diabetic foot ulcers. Rev Esc Enferm U P. 2016 Aug;50(4):628–34.

207. Feitosa MCP, Carvalho AFM de, Feitosa VC, Coelho IM, Oliveira RA de, Arisawa EÂL. Effects of the Low-Level Laser Therapy (LLLT) in the process of healing diabetic foot ulcers. Acta Cir Bras. 2015 Dec;30(12):852–7.

208. Kajagar BM, Godhi AS, Pandit A, Khatri S. Efficacy of low level laser therapy on wound healing in patients with chronic diabetic foot ulcers-a randomised control trial. Indian J Surg. 2012 Oct;74(5):359–63.

209. Kaviani A, Djavid GE, Ataie-Fashtami L, Fateh M, Ghodsi M, Salami M, et al. A randomized clinical trial on the effect of low-level laser therapy on chronic diabetic foot wound healing: a preliminary report. Photomed Laser Surg. 2011 Feb;29(2):109–14.

210. Rosa SSRF, Rosa MFF, Marques MP, Guimarães GA, Motta BC, Macedo YCL, et al. Regeneration of Diabetic Foot Ulcers Based on Therapy with Red LED Light and a Natural Latex Biomembrane. Ann Biomed Eng. 2019 Apr;47(4):1153–64.

211. Huang Q, Yan P, Xiong H, Shuai T, Liu J, Zhu L, et al. Extracorporeal Shock Wave Therapy for Treating Foot Ulcers in Adults With Type 1 and Type 2 Diabetes: A Systematic Review and Meta-Analysis of Randomized Controlled Trials. Can J Diabetes. 2020 Mar;44(2):196-204.e3.

212. Hitchman LH, Totty JP, Raza A, Cai P, Smith GE, Carradice D, et al. Extracorporeal Shockwave Therapy for Diabetic Foot Ulcers: A Systematic Review and Meta-Analysis. Ann Vasc Surg. 2019 Apr;56:330–9.

213. Snyder R, Galiano R, Mayer P, Rogers LC, Alvarez O, Sanuwave Trial Investigators. Diabetic foot ulcer treatment with focused shockwave therapy: two multicentre, prospective, controlled, double-blinded, randomised phase III clinical trials. J Wound Care. 2018 02;27(12):822–36.

214. Wang CJ, Wu RW, Yang YJ. Treatment of diabetic foot ulcers: a comparative study of extracorporeal shockwave therapy and hyperbaric oxygen therapy. Diabetes Res Clin Pract. 2011 May;92(2):187–93.

215. Omar MTA, Alghadir A, Al-Wahhabi KK, Al-Askar AB. Efficacy of shock wave therapy on chronic diabetic foot ulcer: a single-blinded randomized controlled clinical trial. Diabetes Res Clin Pract. 2014 Dec;106(3):548–54.

216. Jeppesen SM, Yderstraede KB, Rasmussen BSB, Hanna M, Lund L. Extracorporeal shockwave therapy in the treatment of chronic diabetic foot ulcers: a prospective randomised trial. J Wound Care. 2016 Nov 2;25(11):641–9.

217. Galiano R, Snyder R, Mayer P, Rogers LC, Alvarez O. Focused shockwave therapy in diabetic foot ulcers: secondary endpoints of two multicentre randomised controlled trials. J Wound Care. 2019 Jun 2;28(6):383–95.

218. Rastogi A, Bhansali A, Ramachandran S. Efficacy and Safety of Low-Frequency, Noncontact Airborne Ultrasound Therapy (Glybetac) For Neuropathic Diabetic Foot Ulcers: A Randomized, Double-Blind, Sham-Control Study. Int J Low Extrem Wounds. 2019 Mar;18(1):81–8.

219. Bajpai A, Nadkarni S, Neidrauer M, Weingarten MS, Lewin PA, Spiller KL. Effects of Non-thermal, Non-cavitational Ultrasound Exposure on Human Diabetic Ulcer Healing and Inflammatory Gene Expression in a Pilot Study. Ultrasound Med Biol. 2018;44(9):2043–9.

220. Yao M, Hasturk H, Kantarci A, Gu G, Garcia-Lavin S, Fabbi M, et al. A pilot study evaluating non-contact low-frequency ultrasound and underlying molecular mechanism on diabetic foot ulcers. Int Wound J. 2014 Dec;11(6):586–93.

221. Ngo O, Niemann E, Gunasekaran V, Sankar P, Putterman M, Lafontant A, et al. Development of Low Frequency (20-100 kHz) Clinically Viable Ultrasound Applicator for Chronic Wound Treatment. IEEE Trans Ultrason Ferroelectr Freq Control. 2019;66(3):572–80.

222. Amini S, ShojaeeFard A, Annabestani Z, Hammami MR, Shaiganmehr Z, Larijani B, et al. Low-frequency ultrasound debridement in patients with diabetic foot ulcers and osteomyelitis. Wounds Compend Clin Res Pract. 2013 Jul;25(7):193–8.

223. Stratmann, B, Costea TC, Nolte C. Effect of Cold Atmospheric Plasma Therapy vs Standard Therapy Placebo onWound Healing in Patients With Diabetic Foot Ulcers A Randomized Clinical Trial - NCT04205942. https://clinicaltrials.gov/show/NCT04205942 [Internet]. 2020; Available from: https://www.cochranelibrary.com/central/doi/10.1002/central/CN-02053349/full

224. Asadi MR, Torkaman G, Hedayati M, Mohajeri-Tehrani MR, Ahmadi M, Gohardani RF. Angiogenic effects of low-intensity cathodal direct current on ischemic diabetic foot ulcers: A randomized controlled trial. Diabetes Res Clin Pract. 2017 May;127:147–55.

225. Asadi M.R., Torkaman G., Mohajeri-Tehrani M.R., Hedayati M. Effects of electrical stimulation on the management of ischemic diabetic foot ulcers. J Babol Univ Med Sci. 2015;17(7):7–14.

226. Kwan RLC, Cheing GLY, Vong SKS, Lo SK. Electrophysical therapy for managing diabetic foot ulcers: a systematic review. Int Wound J. 2013 Apr;10(2):121–31.

227. Kwan RLC, Wong WC, Yip SL, Chan KL, Zheng YP, Cheing GLY. Pulsed electromagnetic field therapy promotes healing and microcirculation of chronic diabetic foot ulcers: a pilot study. Adv Skin Wound Care. 2015 May;28(5):212–9.

228. Elg F, Hunt S. Hemoglobin spray as adjunct therapy in complex wounds: Meta-analysis versus standard care alone in pooled data by wound type across three retrospective cohort controlled evaluations. SAGE Open Med. 2018;6:2050312118784313.

229. Niederauer MQ, Michalek JE, Armstrong DG. A Prospective, Randomized, Double-Blind Multicenter Study Comparing Continuous Diffusion of Oxygen Therapy to Sham Therapy in the Treatment of Diabetic Foot Ulcers. J Diabetes Sci Technol. 2017;11(5):883–91.

230. Yu J, Lu S, McLaren AM, Perry JA, Cross KM. Topical oxygen therapy results in complete wound healing in diabetic foot ulcers. Wound Repair Regen Off Publ Wound Heal Soc Eur Tissue Repair Soc. 2016;24(6):1066–72.

231. Driver VR, Reyzelman A, Kawalec J, French M. A Prospective, Randomized, Blinded, Controlled Trial Comparing Transdermal Continuous Oxygen Delivery to Moist Wound Therapy for the Treatment of Diabetic Foot Ulcers. Ostomy Wound Manage. 2017 Apr;63(4):12–28.

232. Driver VR, Yao M, Kantarci A, Gu G, Park N, Hasturk H. A prospective, randomized clinical study evaluating the effect of transdermal continuous oxygen therapy on biological processes and foot ulcer healing in persons with diabetes mellitus. Ostomy Wound Manage. 2013 Nov;59(11):19–26.

233. Zhang J, Guan M, Xie C, Luo X, Zhang Q, Xue Y. Increased growth factors play a role in wound healing promoted by noninvasive oxygen-ozone therapy in diabetic patients with foot ulcers. Oxid Med Cell Longev. 2014;2014:273475.

234. Liu J, Zhang P, Tian J, Li L, Li J, Tian JH, et al. Ozone therapy for treating foot ulcers in people with diabetes. Cochrane Database Syst Rev. 2015 Oct 27;(10):CD008474.

235. Wainstein J, Feldbrin Z, Boaz M, Harman-Boehm I. Efficacy of ozone-oxygen therapy for the treatment of diabetic foot ulcers. Diabetes Technol Ther. 2011 Dec;13(12):1255–60.

236. Izadi M, Kheirjou R, Mohammadpour R, Aliyoldashi MH, Moghadam SJ, Khorvash F, et al. Efficacy of comprehensive ozone therapy in diabetic foot ulcer healing. Diabetes Metab Syndr. 2019 Feb;13(1):822–5.

237. Razzaghi R, Pidar F, Momen-Heravi M, Bahmani F, Akbari H, Asemi Z. Magnesium Supplementation and the Effects on Wound Healing and Metabolic Status in Patients with Diabetic Foot Ulcer: a Randomized, Double-Blind, Placebo-Controlled Trial. Biol Trace Elem Res. 2018 Feb;181(2):207–15.

238. Momen-Heravi M, Barahimi E, Razzaghi R, Bahmani F, Gilasi HR, Asemi Z. The effects of zinc supplementation on wound healing and metabolic status in patients with diabetic foot ulcer: A randomized, double-blind, placebo-controlled trial. Wound Repair Regen Off Publ Wound Heal Soc Eur Tissue Repair Soc. 2017;25(3):512–20.

239. Soleimani Z, Hashemdokht F, Bahmani F, Taghizadeh M, Memarzadeh MR, Asemi Z. Clinical and metabolic response to flaxseed oil omega-3 fatty acids supplementation in patients with diabetic foot ulcer: A randomized, double-blind, placebo-controlled trial. J Diabetes Complications. 2017 Sep;31(9):1394–400.

240. Mohseni S, Bayani M, Bahmani F, Tajabadi-Ebrahimi M, Bayani MA, Jafari P, et al. The beneficial effects of probiotic administration on wound healing and metabolic status in patients with diabetic foot ulcer: A randomized, double-blind, placebo-controlled trial. Diabetes Metab Res Rev. 2018;34(3).

241. Razzaghi R, Pourbagheri H, Momen-Heravi M, Bahmani F, Shadi J, Soleimani Z, et al. The effects of vitamin D supplementation on wound healing and metabolic status in patients with diabetic foot ulcer: A randomized, double-blind, placebo-controlled trial. J Diabetes Complications. 2017 Apr;31(4):766–72.

242. Armstrong DG, Hanft JR, Driver VR, Smith APS, Lazaro-Martinez JL, Reyzelman AM, et al. Effect of oral nutritional supplementation on wound healing in diabetic foot ulcers: a prospective randomized controlled trial. Diabet Med J Br Diabet Assoc. 2014 Sep;31(9):1069–77.

243. Afzali H, Jafari Kashi AH, Momen-Heravi M, Razzaghi R, Amirani E, Bahmani F, et al. The effects of magnesium and vitamin E co-supplementation on wound healing and metabolic status in patients with diabetic foot ulcer: A randomized, double-blind, placebo-controlled trial. Wound Repair Regen Off Publ Wound Heal Soc Eur Tissue Repair Soc. 2019;27(3):277–84.

244. Basiri R, Spicer M, Levenson C. Nutritional Supplementation Concurrent with Nutrition Education Accelerates theWound Healing Process in Patients with Diabetic Foot Ulcers - NCT04055064. https://clinicaltrials.gov/show/NCT04055064 [Internet]. 2020; Available from: https://www.cochranelibrary.com/central/doi/10.1002/central/CN-01966409/full

245. Bashmakov YK, Assaad-Khalil SH, Abou Seif M, Udumyan R, Megallaa M, Rohoma KH, et al. Resveratrol promotes foot ulcer size reduction in type 2 diabetes patients. ISRN Endocrinol. 2014;2014:816307.

246. Balingit PP, Armstrong DG, Reyzelman AM, Bolton L, Verco SJ, Rodgers KE, et al. NorLeu3-A(1-7) stimulation of diabetic foot ulcer healing: results of a randomized, parallel-group, double-blind, placebo-controlled phase 2 clinical trial. Wound Repair Regen Off Publ Wound Heal Soc Eur Tissue Repair Soc. 2012 Aug;20(4):482–90.

247. Al-Nimer M, Ratha R, Mahwi T. Pentoxifylline improves the quality of life in type-2 diabetes foot syndrome. Pak J Med Sci. 2019 Oct;35(5):1370–5.

248. Janka-Zires M, Almeda-Valdes P, Uribe-Wiechers AC, Juárez-Comboni SC, López-Gutiérrez J, Escobar-Jiménez JJ, et al. Topical Administration of Pirfenidone Increases Healing of Chronic Diabetic Foot Ulcers: A Randomized Crossover Study. J Diabetes Res. 2016;2016:7340641.

249. Zykova SN, Balandina KA, Vorokhobina NV, Kuznetsova AV, Engstad R, Zykova TA. Macrophage stimulating agent soluble yeast β-1,3/1,6-glucan as a topical treatment of diabetic foot and leg ulcers: A randomized, double blind, placebo-controlled phase II study. J Diabetes Investig. 2014 Jul;5(4):392–9.

250. Wang J, Zhu YQ, Li MH, Zhao JG, Tan HQ, Wang JB, et al. Batroxobin plus aspirin reduces restenosis after angioplasty for arterial occlusive disease in diabetic patients with lower-limb ischemia. J Vasc Interv Radiol JVIR. 2011 Jul;22(7):987–94.

251. Gasca-Lozano LE, Lucano-Landeros S, Ruiz-Mercado H, Salazar-Montes A, Sandoval-Rodríguez A, Garcia-Bañuelos J, et al. Pirfenidone Accelerates Wound Healing in Chronic Diabetic Foot Ulcers: A Randomized, Double-Blind Controlled Trial. J Diabetes Res. 2017;2017:3159798.

252. Yingsakmongkol N, Maraprygsavan P, Sukosit P. Effect of WF10 (immunokine) on diabetic foot ulcer therapy: a double-blind, randomized, placebo-controlled trial. J Foot Ankle Surg Off Publ Am Coll Foot Ankle Surg. 2011 Dec;50(6):635–40.

253. Sun X, Jiang K, Chen J, Wu L, Lu H, Wang A, et al. A systematic review of maggot debridement therapy for chronically infected wounds and ulcers. Int J Infect Dis IJID Off Publ Int Soc Infect Dis. 2014 Aug;25:32–7.

254. Elraiyah T, Domecq JP, Prutsky G, Tsapas A, Nabhan M, Frykberg RG, et al. A systematic review and meta-analysis of débridement methods for chronic diabetic foot ulcers. J Vasc Surg. 2016 Feb;63(2 Suppl):37S-45S.e1-2.

255. Wilasrusmee C, Marjareonrungrung M, Eamkong S, Attia J, Poprom N, Jirasisrithum S, et al. Maggot therapy for chronic ulcer: a retrospective cohort and a meta-analysis. Asian J Surg. 2014 Jul;37(3):138–47.

256. Tian X, Liang XM, Song GM, Zhao Y, Yang XL. Maggot debridement therapy for the treatment of diabetic foot ulcers: a meta-analysis. J Wound Care. 2013 Sep;22(9):462–9.

257. Tallis A, Motley TA, Wunderlich RP, Dickerson JE, Waycaster C, Slade HB, et al. Clinical and economic assessment of diabetic foot ulcer debridement with collagenase: results of a randomized controlled study. Clin Ther. 2013 Nov;35(11):1805–20.

258. Motley TA, Gilligan AM, Lange DL, Waycaster CR, Dickerson JE. Cost-effectiveness of clostridial collagenase ointment on wound closure in patients with diabetic foot ulcers: economic analysis of results from a multicenter, randomized, open-label trial. J Foot Ankle Res. 2015;8:7.

259. Brown ML, Tang W, Patel A, Baumhauer JF. Partial foot amputation in patients with diabetic foot ulcers. Foot Ankle Int. 2012 Sep;33(9):707–16.

260. Cheun TJ, Jayakumar L, Sideman MJ, Ferrer L, Mitromaras C, Miserlis D, et al. Short-term contemporary outcomes for staged versus primary lower limb amputation in diabetic foot disease. J Vasc Surg. 2019 Dec 31;

261. Elsherif M, Tawfick W, Canning P, Hynes N, Sultan S. Quality of time spent without symptoms of disease or toxicity of treatment for transmetatarsal amputation versus digital amputation in diabetic patients with digital gangrene. Vascular. 2018 Apr;26(2):142–50.

262. Suh YC, Kushida-Contreras BH, Suh HP, Lee HS, Lee WJ, Lee SH, et al. Is Reconstruction Preserving the First Ray or First Two Rays Better Than Full Transmetatarsal Amputation in Diabetic Foot? Plast Reconstr Surg. 2019;143(1):294–305.

263. Kalantar Motamedi A, Ansari M. Comparison of Metatarsal Head Resection Versus Conservative Care in Treatment of Neuropathic Diabetic Foot Ulcers. J Foot Ankle Surg Off Publ Am Coll Foot Ankle Surg. 2017 Jun;56(3):428–33.

264. Lew E, Nicolosi N, McKee P. Evaluation of Hallux Interphalangeal Joint Arthroplasty Compared With Nonoperative Treatment of Recalcitrant Hallux Ulceration. J Foot Ankle Surg Off Publ Am Coll Foot Ankle Surg. 2015 Aug;54(4):541–8.

265. Vanlerberghe B, Devemy F, Duhamel A, Guerreschi P, Torabi D. [Conservative surgical treatment for diabetic foot ulcers under the metatarsal heads. A retrospective case-control study]. Ann Chir Plast Esthet. 2014 Jun;59(3):161–9.

266. Tardáguila-García A, Sanz-Corbalán I, Molines-Barroso RJ, Álvaro-Afonso FJ, García-Álvarez Y, Lázaro-Martínez JL. Complications associated with the approach to metatarsal head resection in diabetic foot osteomyelitis. Int Wound J. 2019 Apr;16(2):467–72.

267. Maldonado-Rodríguez M, Cajigas-Feliciano Y, Torres-Torres N. Outcomes of osteomyelitis in patients with diabetes: conservative vs. combined surgical management in a community hospital in Puerto Rico. P R Health Sci J. 2011 Jun;30(2):51–7.

268. Dallimore SM, Kaminski MR. Tendon lengthening and fascia release for healing and preventing diabetic foot ulcers: a systematic review and meta-analysis. J Foot Ankle Res. 2015;8:33.

269. Colen LB, Kim CJ, Grant WP, Yeh JT, Hind B. Achilles tendon lengthening: friend or foe in the diabetic foot? Plast Reconstr Surg. 2013 Jan;131(1):37e–43e.

270. Lin J.H., Brunson A., Romano P.S., Pevec W.C., Humphries M.D. Endovascular first treatment is associated with improved amputation-free survival in patients with critical limb ischemia. J Vasc Surg. 2018;67(6):e145.

271. Lo Z.J., Lin Z., Pua U., Quek L.H.H., Tan B.P., Punamiya S., et al. Diabetic foot limb salvage-a series of 809 attempts and predictors for endovascular failure. Vascular. 2016;24(1):61–2.

272. Butt T, Lilja E, Elgzyri T, Apelqvist J, Gottsater A, Engstrom G, et al. Amputation-free survival in patients with diabetic foot ulcer and peripheral arterial disease: Endovascular versus open surgery in a propensity score adjusted analysis. J Diabetes Complications. 2020 Feb 6;107551.

273. Hsu H., Chang C.-H., Lee C.-Y., Huang C.-C., Mark Chiu C.-H., Lin C.-M., et al. A comparison between combined open bypass revascularization and free tissue transfer versus endovascular revascularization and free tissue transfer for lower limb preservation. Microsurgery. 2015 Oct;35(7):518–27.

274. Gentile F, Lundberg G, Hultgren R. Outcome for Endovascular and Open Procedures in Infrapopliteal Lesions for Critical Limb Ischemia: Registry Based Single Center Study. Eur J Vasc Endovasc Surg Off J Eur Soc Vasc Surg. 2016 Nov;52(5):643–9.

275. Hicks C.W., Najafian A., Farber A., Menard M.T., Malas M.B., Black J.H., et al. Below-knee endovascular interventions have better outcomes compared to open bypass for patients with critical limb ischemia. Vasc Med U K. 2017;22(1):28–34.

276. Liistro F, Porto I, Angioli P, Grotti S, Ricci L, Ducci K, et al. Drug-eluting balloon in peripheral intervention for below the knee angioplasty evaluation (DEBATE-BTK): a randomized trial in diabetic patients with critical limb ischemia. Circulation. 2013 Aug 6;128(6):615–21.

277. Liang P, Soden PA, Zettervall SL, Shean KE, Deery SE, Guzman RJ, et al. Treatment outcomes in diabetic patients with chronic limb-threatening ischemia. J Vasc Surg. 2018;68(2):487–94.

278. Deutsch A.J., Jain C.C., Blumenthal K.G., Dickinson M.W., Neilan A.M. Decision-Making in Critical Limb Ischemia: A Markov Simulation. Ann Vasc Surg. 2017;45((Deutsch A.J.; Jain C.C.; Dickinson M.W.) Division of General Internal Medicine, Department of Medicine, Massachusetts General Hospital, Boston, MA, United States):1–9.

279. Butt T, Lilja E, Örneholm H, Apelqvist J, Gottsäter A, Eneroth M, et al. Amputation-Free Survival in Patients With Diabetes Mellitus and Peripheral Arterial Disease With Heel Ulcer: Open Versus Endovascular Surgery. Vasc Endovascular Surg. 2019 Feb;53(2):118–25.

280. Špillerová K, Settembre N, Biancari F, Albäck A, Venermo M. Angiosome Targeted PTA is More Important in Endovascular Revascularisation than in Surgical Revascularisation: Analysis of 545 Patients with Ischaemic Tissue Lesions. Eur J Vasc Endovasc Surg Off J Eur Soc Vasc Surg. 2017 Apr;53(4):567–75.

281. Alexandrescu VA, Brochier S, Limgba A, Balthazar S, Khelifa H, De Vreese P, et al. Healing of Diabetic Neuroischemic Foot Wounds With vs Without Wound-Targeted Revascularization: Preliminary Observations From an 8-Year Prospective Dual-Center Registry. J Endovasc Ther Off J Int Soc Endovasc Spec. 2020 Feb;27(1):20–30.

282. Ambler GK, Stimpson AL, Wardle BG, Bosanquet DC, Hanif UK, Germain S, et al. Infrapopliteal angioplasty using a combined angiosomal reperfusion strategy. PloS One. 2017;12(2):e0172023.

283. Chae KJ, Shin JY. Is Angiosome-Targeted Angioplasty Effective for Limb Salvage and Wound Healing in Diabetic Foot? : A Meta-Analysis. PloS One. 2016;11(7):e0159523.

284. Alexandrescu V., Vincent G., Azdad K., Hubermont G., Ledent G., Ngongang C., et al. A reliable approach to diabetic neuroischemic foot wounds: Below-the-knee angiosome-oriented angioplasty. J Endovasc Ther. 2011;18(3):376–87.

285. Ji D. Evaluation of angiosome-targeted infrapopliteal angioplasty in diabetic critical limb ischemia. Vascular. 2016;24(1):60.

286. Fossaceca R, Guzzardi G, Cerini P, Cusaro C, Stecco A, Parziale G, et al. Endovascular treatment of diabetic foot in a selected population of patients with below-the-knee disease: is the angiosome model effective? Cardiovasc Intervent Radiol. 2013 Jun;36(3):637–44.

287. Söderström M, Albäck A, Biancari F, Lappalainen K, Lepäntalo M, Venermo M. Angiosome-targeted infrapopliteal endovascular revascularization for treatment of diabetic foot ulcers. J Vasc Surg. 2013 Feb;57(2):427–35.

288. Khor BYC, Price P. The comparative efficacy of angiosome-directed and indirect revascularisation strategies to aid healing of chronic foot wounds in patients with co-morbid diabetes mellitus and critical limb ischaemia: a literature review. J Foot Ankle Res. 2017;10:26.

289. Jeon EY, Cho YK, Yoon DY, Kim DJ, Woo JJ. Clinical outcome of angiosome-oriented infrapopliteal percutaneous transluminal angioplasty for isolated infrapopliteal lesions in patients with critical limb ischemia. Diagn Interv Radiol Ank Turk. 2016 Feb;22(1):52–8.

290. Healy A, Farmer S, Pandyan A, Chockalingam N. A systematic review of randomised controlled trials assessing effectiveness of prosthetic and orthotic interventions. PloS One. 2018;13(3):e0192094.

291. Health Quality Ontario. Fibreglass Total Contact Casting, Removable Cast Walkers, and Irremovable Cast Walkers to Treat Diabetic Neuropathic Foot Ulcers: A Health Technology Assessment. Ont Health Technol Assess Ser. 2017;17(12):1–124.

292. Najafi B, Grewal GS, Bharara M, Menzies R, Talal TK, Armstrong DG. Can’t Stand the Pressure: The Association Between Unprotected Standing, Walking, and Wound Healing in People With Diabetes. J Diabetes Sci Technol. 2017;11(4):657–67.

293. Lavery LA, Higgins KR, La Fontaine J, Zamorano RG, Constantinides GP, Kim PJ. Randomised clinical trial to compare total contact casts, healing sandals and a shear-reducing removable boot to heal diabetic foot ulcers. Int Wound J. 2015 Dec;12(6):710–5.

294. Lewis J, Lipp A. Pressure-relieving interventions for treating diabetic foot ulcers. Cochrane Database Syst Rev. 2013 Jan 31;(1):CD002302.

295. Morona JK, Buckley ES, Jones S, Reddin EA, Merlin TL. Comparison of the clinical effectiveness of different off-loading devices for the treatment of neuropathic foot ulcers in patients with diabetes: a systematic review and meta-analysis. Diabetes Metab Res Rev. 2013 Mar;29(3):183–93.

296. Elraiyah T, Prutsky G, Domecq JP, Tsapas A, Nabhan M, Frykberg RG, et al. A systematic review and meta-analysis of off-loading methods for diabetic foot ulcers. J Vasc Surg. 2016 Feb;63(2 Suppl):59S-68S.e1-2.

297. Piaggesi A, Goretti C, Iacopi E, Clerici G, Romagnoli F, Toscanella F, et al. Comparison of Removable and Irremovable Walking Boot to Total Contact Casting in Offloading the Neuropathic Diabetic Foot Ulceration. Foot Ankle Int. 2016 Aug;37(8):855–61.

298. Gutekunst DJ, Hastings MK, Bohnert KL, Strube MJ, Sinacore DR. Removable cast walker boots yield greater forefoot off-loading than total contact casts. Clin Biomech Bristol Avon. 2011 Jul;26(6):649–54.

299. Bus SA, van Netten JJ, Kottink AI, Manning EA, Spraul M, Woittiez AJ, et al. The efficacy of removable devices to offload and heal neuropathic plantar forefoot ulcers in people with diabetes: a single-blinded multicentre randomised controlled trial. Int Wound J. 2018 Feb;15(1):65–74.

300. Potier L, François M, Dardari D. Comparison of a new versus standard removable offloading device in patients with neuropathic diabetic foot ulcers: a French national, multicentre, open-label randomized, controlled trial - NCT01956162. https://clinicaltrials.gov/show/NCT01956162 [Internet]. 2020; Available from: https://www.cochranelibrary.com/central/doi/10.1002/central/CN-02036608/full

301. Chakraborty P.P., Ray S., Biswas D., Baidya A., Bhattacharjee R., Mukhopadhyay P., et al. A comparative study between total contact cast and pressure-relieving ankle foot orthosis in diabetic neuropathic foot ulcers. J Diabetes Sci Technol. 2015 Mar;9(2):302–8.

302. Crawford F, Nicolson DJ, Amanna AE, Martin A, Gupta S, Leese GP, et al. Preventing foot ulceration in diabetes: systematic review and meta-analyses of RCT data. Diabetologia. 2020 Jan;63(1):49–64.

303. Adiewere P, Gillis RB, Imran Jiwani S, Meal A, Shaw I, Adams GG. A systematic review and meta-analysis of patient education in preventing and reducing the incidence or recurrence of adult diabetes foot ulcers (DFU). Heliyon. 2018 May;4(5):e00614.

304. Jiménez S, Rubio JA, Álvarez J, Lázaro-Martínez JL. Analysis of recurrent ulcerations at a multidisciplinary diabetic Foot unit after implementation of a comprehensive Foot care program. Endocrinol Diabetes Nutr. 2018 Oct;65(8):438.e1-438.e10.

305. Monami M, Zannoni S, Gaias M, Nreu B, Marchionni N, Mannucci E. Effects of a Short Educational Program for the Prevention of Foot Ulcers in High-Risk Patients: A Randomized Controlled Trial. Int J Endocrinol. 2015;2015:615680.

306. Ren M, Yang C, Lin DZ, Xiao HS, Mai LF, Guo YC, et al. Effect of intensive nursing education on the prevention of diabetic foot ulceration among patients with high-risk diabetic foot: a follow-up analysis. Diabetes Technol Ther. 2014 Sep;16(9):576–81.

307. Gershater MA, Pilhammar E, Apelqvist J, Alm-Roijer C. Patient education for the prevention of diabetic foot ulcers...Interim analysis of a randomised controlled trial due to morbidity and mortality of participants. Eur Diabetes Nurs. 2011 Sep;8(3):102–107b.

308. Adib-Hajbaghery M, Alinaqipoor T. Comparing the effects of two teaching methods on healing of diabetic foot ulcer. J Caring Sci. 2012 May;1(1):17–24.

309. Hemmati Maslakpak M., Shahbaz A., Parizad N., Ghafourifard M. Preventing and managing diabetic foot ulcers: application of Orem’s self-care model. Int J Diabetes Dev Ctries. 2018;38(2):165–72.

310. Mao X., Mao Q. Effect of motivational interviewing and phased intervention on the self-nursing ability and QOL of patients with a diabetic foot. Int J Clin Exp Med. 2020;13(1):96–103.

311. Sonal Sekhar M., Unnikrishnan M.K., Vijayanarayana K., Rodrigues G.S. Impact of patient-education on health related quality of life of diabetic foot ulcer patients: A randomized study. Clin Epidemiol Glob Health. 2019;7(3):382–8.

312. Jiang L, Mendame Ehya RE. Effectiveness of a Collaborative Nursing Care Model for the Treatment of Patients with Diabetic Foot Disease by Transverse Tibial Bone Transport Technique: A Pilot Study. J Perianesthesia Nurs Off J Am Soc PeriAnesthesia Nurses. 2020 Feb;35(1):60–6.

313. Fernandez MLG, Lozano RM, Diaz MIGQ, Jurado MAG, Hernandez DM, Montesinos JVB. How effective is orthotic treatment in patients with recurrent diabetic foot ulcers? J Am Podiatr Med Assoc. 2013 Aug;103(4):281–90.

314. Rizzo L, Tedeschi A, Fallani E, Coppelli A, Vallini V, Iacopi E, et al. Custom-made orthesis and shoes in a structured follow-up program reduces the incidence of neuropathic ulcers in high-risk diabetic foot patients. Int J Low Extrem Wounds. 2012 Mar;11(1):59–64.

315. Lavery LA, LaFontaine J, Higgins KR, Lanctot DR, Constantinides G. Shear-reducing insoles to prevent foot ulceration in high-risk diabetic patients. Adv Skin Wound Care. 2012 Nov;25(11):519–24; quiz 525–6.

316. Paton J, Bruce G, Jones R, Stenhouse E. Effectiveness of insoles used for the prevention of ulceration in the neuropathic diabetic foot: a systematic review. J Diabetes Complications. 2011 Feb;25(1):52–62.

317. Ulbrecht JS, Hurley T, Mauger DT, Cavanagh PR. Prevention of recurrent foot ulcers with plantar pressure-based in-shoe orthoses: the CareFUL prevention multicenter randomized controlled trial. Diabetes Care. 2014 Jul;37(7):1982–9.

318. Lopez-Moral M, Lazaro-Martinez JL, Garcia-Morales E, Garcia-Alvarez Y, Alvaro-Afonso FJ, Molines-Barroso RJ. Clinical efficacy of therapeutic footwear with a rigid rocker sole in the prevention of recurrence in patients with diabetes mellitus and diabetic polineuropathy: A randomized clinical trial. PloS One. 2019;14(7):e0219537.

319. Bus SA, Waaijman R, Arts M, de Haart M, Busch-Westbroek T, van Baal J, et al. Effect of custom-made footwear on foot ulcer recurrence in diabetes: a multicenter randomized controlled trial. Diabetes Care. 2013 Dec;36(12):4109–16.

320. Cheng Q., Lazzarini P.A., Gibb M., Derhy P.H., Kinnear E.M., Burn E., et al. A cost-effectiveness analysis of optimal care for diabetic foot ulcers in Australia. Int Wound J. 2017 Aug;14(4):616–28.

321. Wu B, Wan X, Ma J. Cost-effectiveness of prevention and management of diabetic foot ulcer and amputation in a health resource-limited setting. J Diabetes. 2018 Apr;10(4):320–7.

322. Cárdenas MK, Mirelman AJ, Galvin CJ, Lazo-Porras M, Pinto M, Miranda JJ, et al. The cost of illness attributable to diabetic foot and cost-effectiveness of secondary prevention in Peru. BMC Health Serv Res. 2015 Oct 26;15:483.
